# The Evidence Base for an Ideal Care Pathway for Frail Multimorbid Elderly: Combined Scoping and Systematic Intervention Review

**DOI:** 10.2196/12517

**Published:** 2019-04-22

**Authors:** Gro Berntsen, Frode Strisland, Kristian Malm-Nicolaisen, Berglind Smaradottir, Rune Fensli, Mette Røhne

**Affiliations:** 1 Norwegian Center for E-health Research University Hospital of North Norway Tromsø Norway; 2 Department of Primary Care Institute of Community Medicine UiT-The Arctic University of Norway Tromsø Norway; 3 SINTEF Digital Oslo Norway; 4 Centre for eHealth University of Agder Grimstad Norway; 5 Research Department Sørlandet Hospital Kristiansand Norway

**Keywords:** systematic review, patient-centered care, delivery of health care, integrated, secondary prevention, risk management

## Abstract

**Background:**

There is a call for bold and innovative action to transform the current care systems to meet the needs of an increasing population of frail multimorbid elderly. International health organizations propose complex transformations toward digitally supported (1) Person-centered, (2) Integrated, and (3) Proactive care (Digi-PIP care). However, uncertainty regarding both the design and effects of such care transformations remain. Previous reviews have found favorable but unstable impacts of each key element, but the maturity and synergies of the combination of elements are unexplored.

**Objective:**

This study aimed to describe how the literature on whole system complex transformations directed at frail multimorbid elderly reflects (1) operationalization of intervention, (2) maturity, (3) evaluation methodology, and (4) effect on outcomes.

**Methods:**

We performed a systematic health service and electronic health literature review of care transformations targeting frail multimorbid elderly. Papers including (1) Person-centered, integrated, and proactive (PIP) care; (2) at least 1 digital support element; and (3) an effect evaluation of patient health and/ or cost outcomes were eligible. We used a previously published ideal for the quality of care to structure descriptions of each intervention. In a secondary deductive-inductive analysis, we collated the descriptions to create an outline of the generic elements of a Digi-PIP care model. The authors then reviewed each intervention regarding the presence of critical elements, study design quality, and intervention effects.

**Results:**

Out of 927 potentially eligible papers, 10 papers fulfilled the inclusion criteria. All interventions idealized Person-centered care, but only one intervention made *what mattered to the person* visible in the care plan. Care coordinators responsible for a whole-person care plan, shared electronically in some instances, was the primary integrated care strategy. Digitally supported risk stratification and management were the main proactive strategies. No intervention included workflow optimization, monitoring of care delivery, or patient-reported outcomes. All interventions had gaps in the chain of care that threatened desired outcomes. After evaluation of study quality, 4 studies remained. They included outcome analyses on patient satisfaction, quality of life, function, disease process quality, health care utilization, mortality, and staff burnout. Only 2 of 24 analyses showed significant effects.

**Conclusions:**

Despite a strong common-sense belief that the Digi-PIP ingredients are key to sustainable care in the face of the silver tsunami, research has failed to produce evidence for this. We found that interventions reflect a reductionist paradigm, which forces care workers into standardized narrowly focused interventions for complex problems. There is a paucity of studies that meet complex needs with digitally supported flexible and adaptive teamwork. We predict that consistent results from care transformations for frail multimorbid elderly hinges on an individual care pathway, which reflects a synergetic PIP approach enabled by digital support.

## Introduction

### The Perfect Storm

The combination of increased longevity, more sensitive diagnostics, and improved treatment contribute to the increasing prevalence of multimorbidity [1-3]. The link between multimorbidity and higher health care spending is well documented, and, most interestingly, the top 10% spenders, who account for two-thirds of all care spending, are dominated by a group of patients with multimorbidity and complex long-term needs [4-6]. Persons with complex long-term needs require coordinated and seamless care from many different providers. However, although other sectors have adopted digital solutions to *glue* fragmented service processes together and enhance both the efficiency and quality of their service, health care lags behind [7]. Following Conway’s law, current information and communication technology infrastructures in health tend to mirror and solidify the silo structures of the organizations they serve [8]. The siloed structure adds to the organizational fragmentation and jeopardizes the information flow [9]. Consequently, the cost and quality of care for persons with complex long-term needs suffer from disruptions, gaps, and duplication of care. Rising expenses, improved single disease treatments, increased proportion of patients with complex long-term needs, and slow adoption of supportive technology are creating a perfect storm threatening the sustainability of our health care systems [10].

### Emerging Responses to New Demands

There is a call for bold and innovative action to reform the current analog, episodic, single disease, profession-centric, and reactive care system to meet the new needs of the population. Health care organizations and researchers in the United States, the World Health Organization, and the European Union are developing roadmaps to deal with the silver tsunami [11-14]. These agree on certain central tenets: we need to improve person-centered care and patient engagement, both because it is the right thing to do and because patient involvement and self-management hold the promise of better and more cost-efficient care [15]. Care fragmentation should be met with integration and seamless digital care plans [16]. Proactive and preventive practices will decrease the need for costly care in both human and economic terms and improve outcomes [17,18]. Last but not least, digital tools are essential to leverage new care models and enable scalability [19-21].

However, the agreement and shared understanding of what *Digitally supported PIP care* is and how it is implemented stop there. Although this strategy seems reasonable, its evidence base is unclear. There are some inspiring success stories around digitally supported large-scale system transformations, notably from the United States with Kaiser Permanente [22,23], South Central Foundation [24], and Veterans’ health administration (VA) [25]. However, these transformations and effects have been tricky to reproduce [26-28], and there is little consensus on the critical elements for success [16,29-32]. Each of the *PIP care* elements has been subject to systematic reviews with mainly encouraging results [16,18,19,31,33-36]. However, each component is often studied on its own, so that the maturity of each element in comparison with other interventions in the same vein and the synergies between them have not been subject to academic study. There is much literature on promising digital solutions, but the large-scale adoption of these tools is slow [37], and there is disillusionment with large-scale electronic health (eHealth) impact [38]. For simplicity in this paper, we will call this triad Digi-PIP care, an abbreviation for *Digi*tally supported *P*erson-centered, *I*ntegrated, and *P*roactive care.

### Research Questions

There is an urgent need to determine whether health services that take a synergistic Digi-PIP care approach to meet the silver tsunami have a documented effect on the triple aims of population health, patient experience, and cost-effectiveness. As care systems vary significantly in the extent they support and operationalize Digi-PIP-care, the review of the documentation is only meaningful if we can get a grasp on the degree of fidelity and maturity of any given implementation.

Our research questions are as follows:

1. How are Digi-PIP care interventions operationalized?
2. How can we capture the maturity of a Digi-PIP intervention?
3. What is the Digi-PIP study methodological quality, and which effects are reported?
4. Does intervention maturity matter for effects?

### Hypotheses and Approach

#### Defining Digitally Supported Person-Centered, Integrated, and Proactive Care

Understanding whether each of the Digi-PIP elements are present or not, and to what degree they are present, is essential to be able to evaluate their effectiveness. In a *drug* model analogy, this would be equivalent to understanding not only if the active drug ingredient is present but also what dosage and administration route is most effective and has fewest side effects. Like in the drug case, we need to grasp the fundamental mechanisms that are responsible for results in the care of patients with complex long-term needs.

However, the individual PIP elements are all poorly defined in the literature. “The term ‘person-centered care’ is used to refer to many different principles and activities, and there is no single agreed definition of the concept.” [39] Likewise, the terms *integrated care* and *proactive care* suffer from the same conceptual ambiguities. Moreover, other terms are synonymous or overlapping with the PIP terms chosen here, such as client-centered care, continuity of care, and anticipatory care [40].

**Figure 1:**
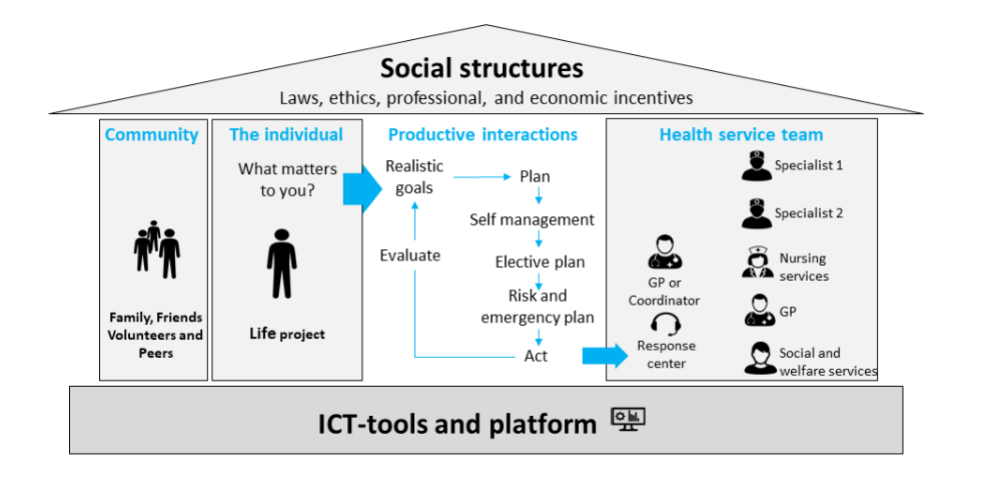
The Person-centered integrated care quality framework. The walls, foundation, and roof symbolize the structural resources. The cyclical care process in the house center consists of exploring what matters to the person and translating this into relevant and realistic goals for care, which feed into a multi-professional care plan. The care team delivers care according to the plan, which is continuously adjusted according to a patient and professional joint evaluation of goal attainment. See text for further explanation. (illustration inspired by House of care by Angela Coulter).

Instead of trying to harmonize definitions from the literature, or inviting professionals to discuss their way to consensus, we have chosen the patient perspective to be our guiding principle. In a previous paper, we show that to patients with complex long-term needs, the essence of care quality was that the care supported their long-term goals, linked to the question: *What matters to you?* [41]. Furthermore, the concept of the individual Patient Pathways as a cycle of Goal setting > Care planning > Care delivery > Care evaluation > Goal adjustment, and so on, made sense to multimorbid patients. All care pathways can be described in these terms, although the fragmented nature of care organization can make this simple pattern difficult to recognize, as each silo will tend to elicit separate parallel individual patient pathways. In Figure 1, the PIP elements are embedded in the 4 stages of the framework.

Below, we describe each of the active elements in PIP care regarding their key characteristics, their care component, and relevant digital support. See also Figure 1, inspired by Coulter [42].

#### Person-Centered Care

*Defining characteristics*: To ensure that care decisions are made in alignment with the person’s answer to the question: *What matters to me?* and that what matters is framed within the scope of realistic, relevant, and safe practices. Common goals have a coordinating effect. The care process is successful when it meets the personal goals [43].

*Care component*: The pathway starts with a sensitive and empathic exploration of *what matters*. A complex care process without a common goal will quickly become a quagmire of competing and poorly coordinated subprocesses, which in the worst case are directly counterproductive. It is essential to include an evaluation of the care process regarding these goals.

*Digital support*: (1) tools that help persons define their goals, such as digital access to pertinent information, shared decision-making tools and interactive self-help tools to think about priorities and goals. Included here is low threshold digital communication with providers, (2) applications that record and share *what matters* and the negotiated goals for care, and (3) interactive tools for a shared evaluation of care according to negotiated goals.

#### Integrated Care

*Defining characteristics*: We define integrated care as a care plan or a multilateral collaboration, which seeks to meet the goals set above, through the coordination of people, information, and physical resources (ie, aids or medications). Care delivery should proceed according to plan. Severe deviations from the plan should lead to a review and reevaluation of the plan, to adjust and set it on course again.

*Care component*: The involved parties should map out the roles, responsibilities, and tasks of all parties, including the person and his or her informal carers linked to the common goal. Although it is possible to address different diagnoses and challenges in separate partial plans, these must come together in a bigger plan, to ensure feasibility within the person’s life and alignment with the overarching goals set by the person. As far as possible and desirable, the care plan should reflect best practice. Monitoring of the care plan delivery is essential to catch gaps in care or derailed plans.

*Digital support*: (1) Tools for interdisciplinary collaboration that identify the care team, roles, and responsibilities, and support communication in virtual teams, including the patient; (2) Tools that support development and sharing of the plan such as decision support and interactive building and update of care plans; (3) Tools that monitor care plan delivery, with alarms in case of critical gaps in care.

#### Proactive Care

*Defining characteristics*: Care which seeks to prevent avoidable outcomes that are costly in both human and economic terms, whenever possible and reasonable.

*Care component* includes both practices that may stabilize and prevent increased risk, such as a comprehensive geriatric assessment [44] and self-management support [45]. It also comprises analyses to identify, monitor, and manage risks factors where early management may prevent clinical deterioration [46]. In population risk management, the idea is to regularly identify persons who might benefit from early intervention [47,48].

*Digital support* is widely expected to transform proactive care. Improved sensor technology and artificial intelligence are promising better ways to detect risk development, support decision making for both patients and professionals in the face of risk, and finally to provide tools for the evaluation of risk intervention [49].

In summary, Digi-PIP care seeks to reduce the risk of clinical crises that are costly in both human and economic terms, mainly through a digitally supported proactive dimension. However, it cannot be stressed enough that Proactive care will fail, if it is not well enough supported by the people who produce the patient pathway: the patient, his or her significant others, and the care professionals. Person-centered care is necessary to understand and integrate the personal agenda into the care plan. The Integrated care elements are necessary to create the conditions in which the relevant competencies are brought together to design the whole person care plan. Only when these 2 elements are in place, can proactive care across conditions and personal agenda be integrated successfully into the care plan activities. The digital support functions as a change agent and is essential to scalability. The triple aim is a product of the synergies of the 3 PIP elements and their digital support.

## Methods

### Theoretical Approach

This paper employs a methodological combination of a scoping review and a systematic intervention review of health service and eHealth research literature. The research literature is the most formal arena for new ideas, discussions, and evaluations of current work toward improved care quality for persons with complex long-term needs.

A scoping study approach helps rapidly identify gaps in existing literature relative to a predefined set of expectations, such as a model or acknowledged challenges in the field and points out areas worthy of further attention [50-52]. A systematic intervention review “...attempts to identify, appraise and synthesize all the empirical evidence that meets pre-specified eligibility criteria to assess the benefits and harms of interventions used in healthcare and health policy” [53].

### The Search Strategy

The search strategy was set out in a protocol document, outlining the intention of the review, the inclusion and exclusion criteria, and the selection and data extraction methods. We give a more detailed outline of our methods, including exact search terms in Multimedia Appendix 1. We knew from previous searches that we were at risk of few included papers. We, therefore, intentionally made inclusion criteria as broad as possible. We searched Ovid MEDLINE, Web of Science, and Scopus. Publications were eligible if they met the following criteria:

- Target population: must include at least a subset of elderly over 60 years with complex care needs. An author’s description of the study population as being frail, multimorbid, or having complex needs was considered sufficient for fulfilling this criterion. We understand frailty in this context as a state of increased biological vulnerability resulting in a reduced ability to cope with stressors [54].
- Intervention: includes all 3 elements defined as:
  - Person-centered care: Paper describes person-centered care as an ideal for care, no definition required.
  - Integrated care: Either a shared care plan or a multidisciplinary team responsible for the cohesive planning and delivery of care or both.
  - Proactive care: early identification of risk, or prevention of risk development at the population or individual level, including self-management support.
- Digital support: any digital technology, supportive of the above intervention components beyond basic electronic health record (EHR) functionality, published after 2000 (to avoid outdated technology).
- Outcome: patient or professional qualitative experience or quantitative measures of the triple aim: (1) patient experience, (2) health outcomes, or (3) cost or benefit ratio.
- Study design: any qualitative or quantitative design, which includes comparisons between situations with or without access to the intervention in either a before-after design or a group comparison.

Exclusion criteria were as follows:

1. Papers that were not original research or had no comparative elements.
2. The study population did not include patients with complex long-term needs.
3. The technology support did not target the Person-centered, Integrated, or Proactive elements. We excluded interventions using a phone, documentation in a general EHR, or shared paper records.

**Figure 2:**
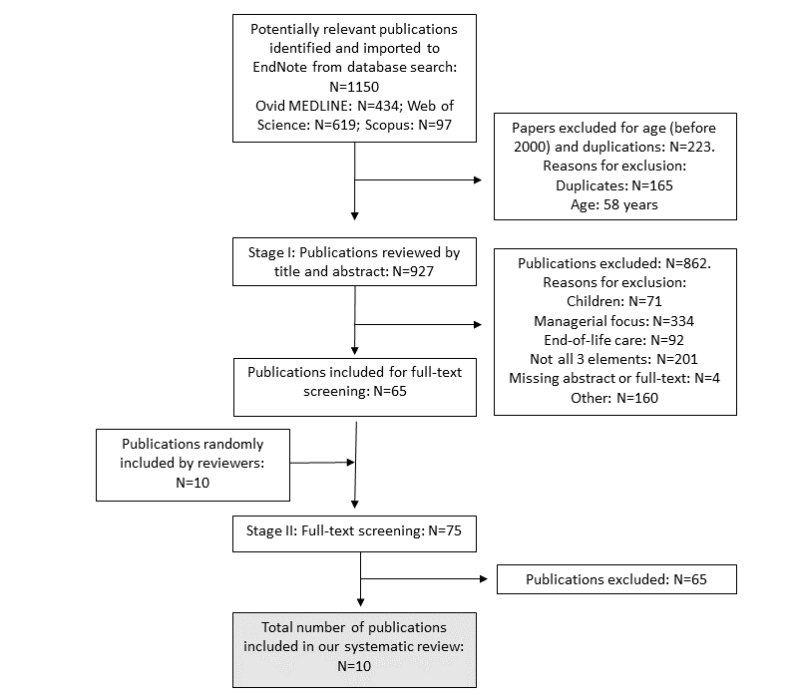
Flowchart of a systematic search and inclusions and exclusions of studies of digitally supported person-centered, integrated, and proactive care (Digi-PIP care) for frail multimorbid elderly. Search finalized in November 2017.

We finalized the search in November 2017. After removal of duplicates, the search identified 927 potentially relevant publications. Moreover, 2 reviewers (KN and KL) independently identified papers that matched the inclusion criteria based on title and abstract. We included 65 publications in the full-text review conducted by FS and GB, and we resolved conflicts between reviewers through discussion until consensus. The study flowchart in Figure 2 shows a final inclusion of 10 publications, originating from 7 studies [55-64].

### Extraction of Data and Analyses

The studies included consist of a sociotechnical intervention expected to improve the individual patient pathway and their corresponding evaluation. Each study, which is our unit of observation, may be described by several papers as shown in Table 1. We used Ritchie’s method for applied policy research, which departs from an a priori thematic framework, inspired by either a policy framework or concepts defined in prior work. In this case, the a priori set of concepts were the Digi-PIP care framework described above. The authors code and review the current text, looking for examples, confirmations, or contradictions to these prior themes [65]. We then add other themes that appear to be central to meaning and interpretation to the framework. We produced a condensed description of each intervention, structured by the PIP elements and their digital support, while also describing other themes central to each paper.

In a secondary, deductive-inductive analysis ad modum Tjora [66], we contrasted the ideas of the primary Digi-PIP care with the intervention descriptions. For each of the PIP care elements, we looked at both care innovation and their parallel digital support. We mapped the presence or absence of capabilities to provide Digi-PIP care and compared and contrasted the subcategories to create mutually excluding key components. We revised the mapping to improve clarity of descriptions and to ensure that we had covered both the theoretical and empirical material. The resulting set of key components reflect ideal maturity when all components are present. Finally, we mapped these key components to a matrix according to their contribution in the generic patient pathway stages: Goals, Plans, Delivery, and Evaluation and by their main active ingredient.

**Table:** 

Included papers’ authors, publication year	Supporting papers	Acronym or short name	Study context	Study population characteristics
Bleijenberg 2016 [55]	Bleijenberg [67]	U-PROFIT (Utrecht PROactive Frailty Intervention Trial)	Netherland, primary care	Randomized controlled trial (RCT) of frail individuals aged >60 years, using screening tools
Blom 2016 [56]	—^a^	ISCOPE (Integrated Systematic Care for Older People)	Netherland, primary care	RCT of Individuals aged >75 years reporting issues in at least 3 of 4 domains in a screening questionnaire
Martin 2012 [63]	—	PaJR (Patient Journey Record system)	Ireland, primary care	At least 1 chronic condition and high health care use last year randomly allocated in intervention and control groups
Boult 2013 [57], 2011 [58]; Boyd 2008 [60], 2007 [59]	Giddens [68]	Guided care	United States, primary care	Cluster randomized trial of persons > 65 years of age and identified as potential high resource users in screening
Council 2012 [61]	—	PCCP (Person-Centered Care Plan)	United States, Dartmouth, clinic with both primary care and hospital services	5 heuristically selected patients with complex care needs. Before after comparison.
Nelson 2014 [64]	Rosland [25], Kearney [69]	PCMH-VA (Person-centered Medical Home, Veterans Health Administration)	United States, Veterans Health Admin, both primary care and hospital services	Observational study of all patients in the Veterans Health Admin system, with subanalyses for persons with chronic conditions
Liss 2011 [62]	Reid [70]	PCMH-GH (Person-centered Medical Home, Group Health)	United States, group health, primary care	Adults with diabetes, hypertension, and/or coronary heart disease at a Patient Centered Medical Home prototype site compared with other sites in Group Health

^a^The paper had no supporting references relevant to this study.

### Assessment of Maturity of the Care System

In an explorative exercise, we used the key components attributed to the 3 active ingredients and their digital support to score each included study regarding maturity. The process of scoring revealed that the interpretation of key components was challenging. The high-level terminology was interpreted differently across authors. Although we were able to arrive at a consensus through dialogue and reflection, it is clear that our scoring system will probably meet the same interpretational challenges in other contexts. Our scores are presented, somewhat tentatively, in Multimedia Appendix 1.

In the next stage, we created the matrix framework, where we translated key system components into system capabilities. A capability is a system’s ability to achieve a desired goal or result and does not specify how the task is solved. These capabilities were then mapped to patient-pathways stages so that it would become more evident what we expect from the system at each stage. We hope this matrix may prompt other researchers to engage in reflection and dialogue about the system capabilities necessary to support a PIP care system.

### Study Design, Quality, and Effects

We used a best-evidence synthesis approach ad modum de Bruin [16] to summarize both study quality and outcomes. Details of the study-quality evaluation can be found in Multimedia Appendix 1. We excluded studies with 2 or fewer quality points in the quality assessment from the outcome’s summary.

Although most of the outcomes were negative, to be parsimonious in our presentation, we have chosen to present all primary outcomes and any secondary outcomes that show a significant effect of the intervention. We present only analyses that are adequately adjusted for baseline biases. We did not attempt to perform a meta-analysis due to the heterogeneity of the contexts, interventions, methodologies, and outcomes. If the intervention reported effects in more than 1 paper, we used data from the latest study.

A short version of the review is presented in Multimedia Appendix 2.

### Ethics and Privacy and Registration of Review

This paper is a literature review. It includes no original information on patients, and there are, therefore, no privacy or ethics concerns requiring board review.

## Results

### Overview of the Empirical Material

Approximately 1.0% (10/927) of the papers identified in the digital search (10 papers representing 7 interventions of 927 potential papers) were included in the final review. We present the intervention context and study population in Table 1.

### How Are Digitally Supported Person-Centered, Integrated, and Proactive Care Interventions Operationalized?

#### Person-Centered Care

All studies claimed to adhere to and acknowledge the long-term aspect of the care process. The degree to which they document that personal goals make an impact on care decisions varied from the mention of Person-centered care as an ideal for care at one end of the scale to a documented impact on care plans and evaluation at the other.

Only the Person-Centered Care Plan (PCCP) study reached the highest possible maturity score on Person-centered maturity. They document how they understand person-centeredness as a journey undertaken by the patient and the surrounding team. The goal in the PCCP is “...to create negotiated goals that incorporate the values of the patient and the healthcare team into a mutually agreed upon explicit action plan.” Council demonstrates how the PCCP identifies the care team and distributes responsibilities for goals and tasks, including the goals for which the patient is responsible. The care plan outlines what to do in an emergency. The PCCP is digital, which means it is interactive, updated, and shared among those providers that have access to the same EHR [61]. It is not clear if the patient has access to the PCCP.

Integrated Systematic Care for Older People (ISCOPE) mentions goal setting together with the patient: “...care should be provided proactively to set and prioritize goals together with the patient and to empower the patient to reach these goals.” [56]. However, ISCOPE describes the care plan as follows: “Each patient received a geriatric assessment, a comprehensive care plan, evidence-based primary care with proactive follow-up of chronic conditions, coordination of the efforts of health professionals across all health care settings, and facilitated access to community resources.” [56]. This description leaves open how the patient goals were incorporated or affected the plan.

The other 5 studies described Person-centered care in terms of patient involvement and engagement. However, they did not document how *what matters* nor how patient needs, values, or preferences were linked to planning and decision making [13,55,57,62-64]. There were no systems that offered digital tools for goal setting, such as shared decision making. No papers mention patient’s evaluation of goals or patient-reported outcome measures (PROMs), neither on paper nor digitally.

#### Integrated Care

##### Care Plan and Care Manager or Team

All the papers highlight fragmentation of care as a significant challenge and explain how their intervention supports seamless care. Evidence-based and shared care plans are core to the integrated care effort [55-57,61,62]. Digital tools to make the plan, such as evidence-based decision support and health maintenance reminders, were available in the Person-Centered Medical Home, Group Health study [62]. No study mentioned disease-specific paper-based or digital standardized care pathways as a building block for personalized care plans. The PCCP, ISCOPE, and the Person-Centered Medical Home, Veterans Health Administration (PCMH-VA) studies shared the care plan electronically with other providers in a common EHR [57,61,64]. However, it is unclear to which degree the plan is available electronically to parties outside the organization, such as the patient.

In addition to a care plan, 5 of the studies dedicated extra resources to the development, delivery, recruitment of external resources, and follow-up of the care plan [55-60,62,64]. This could take the form of *a case manager* [55-57,63], or a broader, multidisciplinary, *primary care team* [56,57,62]. Except for shared care plans, as described above, we could not find mention of digital support for team communication, such as group video meetings or asynchronous group chats.

##### Care Delivery

Effect of a care plan is contingent on its actual delivery, but no system monitored care plan delivery systematically. Moreover, 2 studies observed patient progress "by monthly monitoring of symptoms and adherence [57] and measurement-based assessment of progress with facilitation of treatment changes" [69]. Council’s PCCP shows both who is responsible for a given action and when it is expected to be done [61]. We found no mention of how care plans were coupled to resource allocation, neither organizationally nor digitally. We found no study where workflow optimization facilitated the transition from planning to delivery of care in any study.

#### Proactive Care

There are 2 main approaches to proactive care: self-management support and risk and emergency management.

##### Self-Management Support

Self-management support improves the person’s capacity to maintain health and well-being, and it simultaneously strengthens self-agency. Both the PCCP and the Guided Care studies describe how the care plan includes those actions that the patient is responsible for [57,61]. The PCCP study mapped patient strengths and used this as a basis for the self-management support plan. Self-management support also consisted of motivational interviews, patient education, activation workshops, and behavioral counseling [57,62,64,69].

A total of 3 papers did not report any activities on self-management support [55,56,63]. The interventions do not mention widely available self-help applications or self-help communities as tools for self-management. No papers linked patients to community resources.

##### Risk and Emergency Management

Risk assessment strategies identify and act upon early impactable risk, rather than wait for the clinical crisis. Risk assessment at the individual level was an element in 6 of the 7 studies. The Patient Journey Record system (PaJR) study used lay care guides to keep in touch with patients at high risk for hospitalization. After each conversation, a natural language analysis of the written synopsis would estimate the risk of hospitalization [63]. Digital health analytics from GP-EHR or insurance data identified frailty or risk for high care expenses in the Utrecht PROactive Frailty Intervention Trial (U-PROFIT) [55] and Guided care studies [57], and, in the latter study, also regular follow-up phone calls to assess changes in risk [57]. In the PMCH-GH intervention, patients filled an eHealth risk assessment form [62]. In the ISCOPE study, the risk was assessed once through a questionnaire sent to elderly citizens, without the use of digital tools. In the PMCH-VA study, they mapped unhealthy lifestyle habits [64]. Only the PCCP study had no risk assessment included [61].

The risk identification was linked to a range of *actions*. In the PaJR study, the lay care guide would notify the appropriate usual care service, such as the general practitioner or a case manager [63]. In the U-PROFIT [55], ISCOPE [56], and Guided Care studies, a nurse or GP-nurse team would develop a comprehensive and evidence-based geriatric care plan [55-57]. The PMCH-VA self-reported risk assessment is not described to be directly linked to any intervention activity, except for general training in lifestyle coaching and motivational interviewing to all clinical employees [25,69]. Remote telehealth follow-up from registered nurses was mentioned as an opportunity in some VA facilities [25]. No studies described digital decision support for alarm situations.

Only the PCCP plan summarized the emergency measures agreed by both patient and providers [61]. Low-threshold one point of contact, such as virtual video contacts for emergencies and questions, was emphasized in the VA and Group health [62,64]. Other papers outlined no emergency contingency plans [55-57,62-64].

### The Maturity of Digitally Supported Person-Centered, Integrated, and Proactive Care Models

We have conceptualized Digi-PIP care as a set of characteristics systematically included in the individual Patient Pathway in support of the 4 generic stages of a patient pathway [41]. Each key component is described in terms of the capability the system offers. The more elements present, the higher the maturity of the care system in question (see Table 2).

Although all the included papers addressed the 3 active ingredients and some form of digital support, it was clear that none of the interventions succeeded in giving equal focus to all elements.

The digital support, particularly, was marginal and far less advanced than what is considered state of the art in research projects addressing only 1 of the PIP digital axis. PCMH-VA had the highest total digital score, with a patient portal, low-threshold e-visits, a shared care plan, and telemonitoring services. They were far from sporting a full suite of eHealth services that would both leverage and scale their PCMH approach. All other studies lacked digital support in at least 1 PIP elements. We believe that a genuinely sociotechnical design of PIP care, where technology supports and replaces analog services, is still somewhere in the future.

Even when the care packages are complex, the studies do not explicitly acknowledge the interwoven dependencies between the PIP components. There is some understanding that all Digi-PIP ingredients are essential. For instance, in U-PROFIT study, the main focus was on integrated care, whereas the person-centered and proactive components were considered integral parts of the overarching comprehensive care plan [55].

However, the focus on the unbroken chain of care events that lead from intervention to the desired outcome is not always present. For example, care outcomes require loyalty in care delivery to the care plan. Impressive as the service redesign efforts in the Guided Care study is, the Guided care nurse is *grafted* onto the usual care system in a supportive role. If the carefully crafted Guided care plan does not fit with the agenda of the usual providers, the guided care plan may be set aside [59]. In the VA study by Nelson, care professionals were encouraged to explore *what matters* to patients and used motivational interviewing to do so. The integrated care planning did not seem to build on the goals brought forward by the patients. Moreover, the proactive component included mostly primary preventions such as smoking cessation but did not include individualized risk monitoring and management, which is presumably more efficient. The clever monitoring of a high-risk situation is only effective if the response to deal with that risk is adequate [25,69]. We found similar weak links in the *chain of success* in all of the 7 studies.

**Table 2:** Key care and digital components, described in terms of the capabilities they offer in support of the person-centered, integrated, and proactive care. We have mapped each PIP-element to statements of care system capability for each of the 4 generic stages of an individualized patient pathway.

Care components		Goals	Plans	Delivery	Evaluation
**The person-centered care system…**					
	Care	...declares Person-centered care as an ideal and explores “what matters to me?” and “patient values, needs, and preferences.”	...uses “What matters to me?” to negotiate realistic goals and create a care plan.	...includes patient capabilities aligned with “What matters to me?” in care delivery.	...asks for patient feedback/ PROMs^a^
	Digital support	...offers access to digital health information/ electronic health record and supports the formulation of “what matters to me?”	...offers digital sharing of: What my carers should know about me.	...includes the patient in virtual care delivery and team exchanges.	...encourages digital feedback from patients, including PROMs.
**The integrated care system…**					
	Care delivery	...identify condition- or function-specific goals that support “what matters.”	...combines condition/function-specific pathways into a whole person care plan for all conditions.	...allocates resources to care plan, to show who does what when.	...follow up to identify needs for adjustment of care plans or delivery.
	Digital support	...digitally identifies potential professionals to contribute to care plan development aligned with “what matters to me?”	...provide tools to build a personalized digital evidence-based care plan, with workflow optimization to show: who does what when.	...shares the care plan digitally across providers and offers tools for virtual team communication (video, messages, and chat).	...triggers an alarm in case of gaps in critical care delivery.
**The proactive care system…**					
	Care delivery	...identifies high-risk subpopulations, their individual high-risk scenarios over time and aligns focus on risk with ”What matters to me?“	...supports risk monitoring, self-managed or professional follow-up, in alignment with ”What matters to me?“	...offers low threshold response (self-managed, office or home visits) to uncertainties, emergencies, and alarms.	...learns and adjusts goals and plans in light of undesired events and ”What matters to me?“
	Digital support	...offers an algorithm-based risk-stratification tool to identify high-risk populations and their individual risk scenarios over time.	...offers personal digital health apps and sensors that monitor risk and provide digital contingency plans in case of uncertainty, emergencies, or alarms.	...provides digital decision support and low-threshold e-visits in case of uncertainty, emergencies, or alarms.	...is a learning health care system improves prediction and action plans in light of undesired events.

^a^PROMs: patient-reported outcome measures.

### Study Design Quality and Effects

In compliance with de Bruin’s methodology [16], we include only studies with a quality score of 3 or more in our effect report, which left us with 4 studies. We present the quality scores in Multimedia Appendix 1. The included studies are 3 cluster randomized trials at General practice level (U-PROFIT, ISCOPE, and Guided Care) reporting on patient-level data, whereas the fourth is an observational study utilizing aggregated measures at clinic level in the PMCH-VA study.

The U-PROFIT, ISCOPE, Guided Care, and PCMH-VA studies present 8, 3, 7, and 6 outcome analyses, respectively, 24 in all. Outcomes lie within areas of patient satisfaction, quality of life, function, disease process quality, health care utilization, mortality, and staff burnout. Of these, only the emergency department visits in Nelson’s study from the VA and the home-care visit frequency in Guided Care showed clear and clinically meaningful significant positive effects (see Table 3) [57,64]. The U-PROFIT study showed a slower functional decline in the intervention group compared with controls, but the clinical significance was deemed uncertain (Table 3) [55]. None of the other analyses showed significant effects of the intervention.

This study cannot answer the question of whether intervention maturity matters, because the number of high-quality studies is not large enough to support a correlation analysis between maturity and outcomes.

**Table 3:** Selected outcomes in 4 high-quality studies of *digitally supported Person-centered, Integrated, and Proactive care* (digi-PIP-care) for frail multimorbid elderly. All primary outcomes and any positive secondary outcomes analyses are shown. Negative secondary analyses not presented.

Paper	Outcome measure	Patient or clinics	N	Effect intervention	Effect control	Ratio Intervention/ Control	*P* value
Utrecht PROactive Frailty Intervention Trial [55]	Katz 15 scores at 6 months. Range 0-15, lower score is better	P^a^	2754	1.7	1.7	0.97	Not significant
	Katz 15 scores at 12 months. Range 0-15, lower score is better	P	2489	1.9	2.0	0.92	.03
Integrated Systematic Care for Older People [56]	12 months follow-up, change in quality of life, Cantril’s ladder (range 0-10, higher is better)	P	842	−0.2	−0.2	1.00	.82
	12 months follow-up, Delta Groningen Activities Restriction Scale (range 18-72, lower score is better).	P	842	2.9	3.5	0.83	.30
Guided Care [57]	Functional health Short Form 36, higher score is better	P	477	36.1	37.5	0.96	Not significant
	Home health care episodes	P	477	0.9	1.3	0.71	<.05
Person-centered Medical Home-Veterans Health Administration [64]	Emergency Department visits per 1000 patients per year (secondary outcome)	Clinic	913	188	245	0.77	<.001

^a^P: Patient.

## Discussion

### Summary of Findings

We identified 927 potentially eligible papers, but after full-text review, we included only 10 papers.

The PIP elements were supported to a varying degree. Person-centeredness was an ideal for care, but only one intervention made *what mattered to the person* visible throughout the care plan. The studies counter care fragmentation through a whole-person care plan and the engagement of care-coordinating case managers or multiprofessional teams. Care delivery according to plan seems to be taken for granted as none monitor system delivery of care plans. Although papers mention self-management support and emergency plans are mentioned, risk identification and management are the main proactive strategy. No studies suggested workflow optimization or patient-reported outcomes.

The most prominent digital support of the PIP elements was risk stratification tools. Second, 3 providers supported the sharing of care plans in the EHR.

The chain of care is only as strong as its weakest link. The maturity matrix made it possible to identify several potential breaks or weaknesses in the chain of success. The most common weaknesses were:

1. A lack of documentation that *what mattered to the person* was also brought to bear on care plans and delivery.
2. That planned care plan was actually delivered.
3. That risk identification schemes were coupled with adequate responses from the care system.
4. Finally, there are feedback loops that support learning and adjustment of the PIP dimensions of care.

We included 4 studies in our summary of effects after methodological quality assessment. Moreover, 2 of the 24 analyses in 4 studies reported modest positive outcomes with reductions in emergency care utilization and home health visits.

### The Invisible Sociotechnical Care Process

The specialization of health care remains both its biggest asset and weakness. Systems theory has shown long ago that when systems grow, they tend to specialize. If specialization is not coupled with centralized coordination, the organization’s ability to deliver its end product will be crippled [71]. In other services (banking, the tourist industry, publishing, and e-commerce), digital tools are the glue that allows all the involved parties, irrespective of professional and organizational affiliation, to work effectively together. The digitally supported processes organize people, information, and things into a value chain for the patient. The need to share goals and plans, understand roles and tasks, and learn to support each other collectively is the same in health care as in other industries. The digital infrastructure is lagging. Strict privacy rules, lack of e-governance structures, lack of interoperability standards, and lack of business models for eHealth vendors are acknowledged barriers [72]. The infrastructure is slowly coming into place, but not nearly fast enough to catch up with other industries.

### Weaknesses in the Chain of Success

We structured this review according to the Digi-PIP care framework and a maturity evaluation. As noted, there is an abundance of literature and reviews supporting each of the PIP elements alone [15-17,31,45,49]. However, all of the reviews also note the heterogeneity of the interventions and the lack of consistency in results. In this light, we hypothesize that the wide variation of effects in Digi-PIP care interventions are attributable to the weaknesses in the care system’s implementation and understanding of *chains of success*: The current efforts are not bringing us closer to the triple aim. So what needs to change? We claim that approaches to date have failed to address the full complexity of the problem, both in the health care system design itself and in the corresponding scientific intervention with outcome analysis approaches.

### Complex Adaptive Systems

Scientists are taught to narrow down and examine *one factor at a time*, and the randomized controlled trial (RCT) is the golden standard of how to ascertain the effect of the single factor. RCTs are designed for hypothesis testing, that is, A works on B to produce C with mechanism D, when context X, Y, and Z are stable. However, this requires the researcher to have a reasonable hypothesis on how the *one factor* works, under what conditions it works, and to keep all other conditions stable for the duration of the experiment. The RCT has, however, become a *test tool* for complex interventions in complex settings, where the assumptions under scrutiny are unclear. The validity of such an RCT is low because there is no clear hypothesis. If we test an antibiotic on cases of viral infection, it will look as if the antibiotic did not work. The truth is that we did not understand the mechanism of action well enough to design the study correctly. We need to move beyond the RCT and use other tools to understand how to improve the outcomes of the complex and fluid social processes of health care.

### A Good Enough Vision

Complexity theory [73] and quality improvement theory [74,75] prescribe a different set of methods to understand, improve, and predict outcomes in complex adaptive systems [76]. Although it is outside our scope to describe complex adaptive systems in full, there are some important points worth reflecting over in light of our review. Complexity theory predicts that a linear plan where method X leads to outcome A will not be successful in a complex adaptive systems. This is because unknowable factors will frustrate the method X in a proportion of the cases.

Instead of placing all faith in method X, the intervention will need to include a way to detect and manage challenges as professionals become aware of them. In complex adaptive systems, one moves toward a goal first and foremost by creating a good enough vision of what the goal is. In Digi-PIP care, we believe that vision is a negotiated and realistic set of personalized goals aligned with *what matters* to the person. The next step is to provide frontline professionals with an array of tools and checkpoints that are useful in the creation of an individualized road map toward the personalized goals. In Digi-PIP care, this will be a set of professional knowledge and skill sets, more or less evidence-based, more or less proactive, assembled in the integrated care team. Depending on the context, the agents must apply their knowledge and experience to choose a way forward. If they are closing in on the goal, they stay on course. If the goal is slipping, they must reevaluate and adjust. In Digi-PIP care, that will amount to evaluation not only of the person’s overarching goals, which may be long term and difficult to maneuver by. Choosing proximal and sensitive subgoals that support the long-term goals may also be useful. The capabilities identified in the maturity matrix may serve as tools to create subgoals in each case. Adopting a *chain of success* way of thinking to understand which factors must line up to create a desired outcome in the unique case can be productive. For instance, the lack of a care plan is an obvious impediment to seamless, coherent care delivery. However, a care plan does not translate into care delivery by magic. Assuring care delivery according to plan is a part of the challenge to reach the desired effect. A close dialogue with the patient throughout the process will help this continuous guiding evaluation to take place. There also needs to be a sensitivity to changing goals, as insights and contexts change for the patient and the team.

### Improving Outcomes in Complex Adaptive Systems

The researchers who work in improving outcomes in complex adaptive systems will be working with questions such as How can we help formulate good enough goals and subgoals, that are also observable, and provide process guidance?; What are areas of knowledge, skills, and tools essential to make available to enable professionals to invent proper processes?; Are there standardized components that can be plugged into and tailored to the individual pathway?; and How can we help frontline professionals hypothesize and modulate the chain of events that will lead to success in each case?

### The Validity of Our Work—Strengths and Limitations

We have done systematic searches in the 3 largest literature databases covering the health and eHealth field. A librarian trained in building complex searches conducted the search. Pairs or triplets of authors performed all the steps in the screening of papers, data extraction, and grading of papers. Author-pairs discussed disagreements until they reached consensus, or, if it concerned a matter of principle, the entire author group addressed the issue. We defined rather broad inclusion criteria so that we should not inadvertently exclude studies that might bring forth new knowledge. The authors were, with one exception, senior researchers at the professor or associate professor level with long track records in the health and eHealth fields. These are the strengths of the study, which ensure that we have indeed identified the current published knowledge base regarding Digi-PIP care.

Given the extensive activity in this field and the many large enterprises underway in this area, we were surprised by the meager catch and by how old many of the papers were. We believe that the small number of articles indicates that this is an area that many researchers find too complicated to bring into a viable research model. Those organizations that are making progress in this field do so without publishing their interventions, the digital solutions, and their results. There may well be significant experiential knowledge in the field that we do not capture in this review.

### Conclusions

The research literature is permeated with *common sense* argumentation that each of the Digi-PIP elements is important. However, many hardworking and extremely talented health care scientists have to date chosen to address just a subset of the elements in their research. We identified only 10 papers from 7 interventions reporting on studies encompassing all elements together, and they all report a limited effect. The general lack of effects on the triple aim from both subset approaches and all element approaches until now is also disappointing for the research and practice field. We argue that it is now time to rethink our approaches to health care innovation by acknowledging the patient voice and the inherent system complexities.

It is not enough to provide a care plan that seems sensible to the providers. It must also be *owned* by the person himself or herself, who is the crucial resource, enabler, and guide. It is not enough to make a care plan unless there is also considerable devotion to the delivery of the care plan and attention to whether its objectives are met. It is not enough to be proactive in an elective care plan if one does not also monitor risk for the impending crisis and provide an emergency plan, which can help avoid it. Finally, it is probably not possible to address these complex processual challenges with regular EHR, paper, and phone. It is time to say that the health care sector is under-digitized and that the lack of appropriate digital support is a barrier for PIP-care and costs both patients and professionals dearly.

We believe that a reductionist scientific methodology is blocking the way forward. We need to embrace the problem-solving methods suited for the improvement of outcomes in complex adaptive systems. Researchers need to embrace questions such as How do we formulate good enough process guiding goals?; How does a professional formulate a rationale for adjustment of a process?; and What are the generalizable components of *individualized* pathway creation?

We predict that research will not show consistent results from care transformation for persons with complex long-term needs until all 3 PIP care elements are successfully and flexibly implemented with digital support. We need a chain of success thinking in the work of creating patient pathways. The art of high-quality care is to invent a road as it is being walked, toward *what matters to the person* in every pathway for patients with complex long-term needs.

## Appendix

Multimedia Appendix 1 More detail on methods, and literature review search strings.

Multimedia Appendix 2 The short version: The evidence base for an ideal care pathway for frail multi-morbid elderly: A combined scoping and systematic intervention review.

## Abbreviations

Digi-PIP: Digitally supported Person-centered, Integrated, and Proactive care
eHealth: electronic health
EHR: electronic health record
ISCOPE: Integrated Systematic Care for Older People
PaJR: Patient Journey Record system
PCCP: Person-Centered Care Plan
PCMH-VA: Person-Centered Medical Home, Veterans Health Administration
PIP: Person-centered, Integrated, and Proactive
PROMs: patient-reported outcome measures
RCT: randomized controlled trial
U-PROFIT: Utrecht PROactive Frailty Intervention Trial
VA: Veterans Health Administration

## References

[1] Afshar S, Roderick P, Kowal P, Dimitrov B, Hill A, Hoque MN, Pecotte B, McGehee MA (2017). Global Patterns of Multimorbidity: A Comparison of 28 Countries Using the World Health Surveys. Applied Demography and Public Health in the 21st Century.

[2] Violan C, Foguet-Boreu Q, Flores-Mateo G, Salisbury C, Blom J, Freitag M, Glynn L, Muth C, Valderas JM (2014). Prevalence, determinants and patterns of multimorbidity in primary care: a systematic review of observational studies. PLoS One.

[3] van Oostrom SH, Gijsen R, Stirbu I, Korevaar JC, Schellevis FG, Picavet HS, Hoeymans N (2016). Time trends in prevalence of chronic diseases and multimorbidity not only due to aging: data from general practices and health surveys. PLoS One.

[4] Wang L, Si L, Cocker F, Palmer AJ, Sanderson K (2018). A systematic review of cost-of-illness studies of multimorbidity. Appl Health Econ Health Policy.

[5] Zulman DM, Pal CC, Wagner TH, Yoon J, Cohen DM, Holmes TH, Ritchie C, Asch SM (2015). Multimorbidity and healthcare utilisation among high-cost patients in the US Veterans Affairs Health Care System. BMJ Open.

[6] Heiberg I (2015). High utilisation patients in somatic specialist healthcare in Northern Norway [Storforbrukere av somatisk spesialisthelsetjeneste i Helse Nord. SKDE].

[7] Ossebaard HC, Van Gemert-Pijnen L (2016). eHealth and quality in health care: implementation time. Int J Qual Health Care.

[8] Conway M (1968). How do committees invent?. Datamation.

[9] Coiera E (2004). Four rules for the reinvention of health care. Br Med J.

[10] Berwick DM, Nolan TW, Whittington J (2008). The triple aim: care, health, and cost. Health Aff (Millwood).

[11] Patient-Centered Primary Care Collaborative. (2015). Patient-Centered Primary Care Collaborative.

[12] (2016). World Health Organization.

[13] Rich E ELM, Lipson D, Libersky J, Parchman M (2012). Patient-Centered Medical Home.

[14] Joint Action CHRODIS (2017). http://chrodis.eu/wp-content/uploads/2018/01/multimorbidity_care_model.pdf.

[15] Coulter A, Entwistle VA, Eccles A, Ryan S, Shepperd S, Perera R (2015). Personalised care planning for adults with chronic or long-term health conditions. Cochrane Database Syst Rev.

[16] de Bruin SR, Versnel N, Lemmens LC, Molema CC, Schellevis FG, Nijpels G, Baan CA (2012). Comprehensive care programs for patients with multiple chronic conditions: a systematic literature review. Health Policy.

[17] Steventon A, Bardsley M, Billings J, Dixon J, Doll H, Hirani S, Cartwright M, Rixon L, Knapp M, Henderson C, Rogers A, Fitzpatrick R, Hendy J, Newman S, Whole System Demonstrator Evaluation Team (2012). Effect of telehealth on use of secondary care and mortality: findings from the Whole System Demonstrator cluster randomised trial. Br Med J.

[18] Hallberg IR, Kristensson J (2004). Preventive home care of frail older people: a review of recent case management studies. J Clin Nurs.

[19] Ekeland AG, Bowes A, Flottorp S (2010). Effectiveness of telemedicine: a systematic review of reviews. Int J Med Inform.

[20] Hersh WR, Totten AM, Eden KB, Devine B, Gorman P, Kassakian SZ, Woods SS, Daeges M, Pappas M, McDonagh MS (2015). Outcomes from health information exchange: systematic review and future research needs. JMIR Med Inform.

[21] Woods SS, Evans NC, Frisbee KL (2016). Integrating patient voices into health information for self-care and patient-clinician partnerships: Veterans Affairs design recommendations for patient-generated data applications. J Am Med Inform Assoc.

[22] Ham C, York N, Sutch S, Shaw R (2003). Hospital bed utilisation in the NHS, Kaiser Permanente, and the US Medicare programme: analysis of routine data. Br Med J.

[23] Feachem RG, Sekhri NK, White KL (2002). Getting more for their dollar: a comparison of the NHS with California's Kaiser Permanente. Br Med J.

[24] Driscoll DL, Hiratsuka V, Johnston JM, Norman S, Reilly KM, Shaw J, Smith J, Szafran QN, Dillard D (2013). Process and outcomes of patient-centered medical care with Alaska Native people at Southcentral Foundation. Ann Fam Med.

[25] Rosland AM, Nelson K, Sun H, Dolan ED, Maynard C, Bryson C, Stark R, Shear JM, Kerr E, Fihn SD, Schectman G (2013). The patient-centered medical home in the Veterans Health Administration. Am J Manag Care.

[26] Busse R, Stahl J (2014). Integrated care experiences and outcomes in Germany, the Netherlands, and England. Health Aff (Millwood).

[27] Hughes G (2017). New models of care: the policy discourse of integrated care. PPP.

[28] Salisbury C, Man M, Bower P, Guthrie B, Chaplin K, Gaunt DM, Brookes S, Fitzpatrick B, Gardner C, Hollinghurst S, Lee V, McLeod J, Mann C, Moffat KR, Mercer SW (2018). Management of multimorbidity using a patient-centred care model: a pragmatic cluster-randomised trial of the 3D approach. Lancet.

[29] Scott IA (2008). Chronic disease management: a primer for physicians. Intern Med J.

[30] Beswick AD, Rees K, Dieppe P, Ayis S, Gooberman-Hill R, Horwood J, Ebrahim S (2008). Complex interventions to improve physical function and maintain independent living in elderly people: a systematic review and meta-analysis. Lancet.

[31] Lemmens LC, Molema CC, Versnel N, Baan CA, de Bruin SR (2015). Integrated care programs for patients with psychological comorbidity: a systematic review and meta-analysis. J Psychosom Res.

[32] Valentijn PP, Schepman SM, Opheij W, Bruijnzeels MA (2013). Understanding integrated care: a comprehensive conceptual framework based on the integrative functions of primary care. Int J Integr Care.

[33] Coulter A, Ellins J (2007). Effectiveness of strategies for informing, educating, and involving patients. Br Med J.

[34] Stellefson M, Dipnarine K, Stopka C (2013). The chronic care model and diabetes management in US primary care settings: a systematic review. Prev Chronic Dis.

[35] Huntley AL, Thomas R, Mann M, Huws D, Elwyn G, Paranjothy S, Purdy S (2013). Is case management effective in reducing the risk of unplanned hospital admissions for older people? A systematic review and meta-analysis. Fam Pract.

[36] Tenforde AS, Hefner JE, Kodish-Wachs JE, Iaccarino MA, Paganoni S (2017). Telehealth in physical medicine and rehabilitation: a narrative review. PM R.

[37] Kaplan B, Harris-Salamone KD (2009). Health IT success and failure: recommendations from literature and an AMIA workshop. J Am Med Inform Assoc.

[38] Greenhalgh T, Russell J (2010). Why do evaluations of eHealth programs fail? An alternative set of guiding principles. PLoS Med.

[39] (2014). The Health Foundation.

[40] Ehrlich C, Kendall E, Muenchberger H, Armstrong K (2009). Coordinated care: what does that really mean?. Health Soc Care Community.

[41] Berntsen G, Høyem A, Lettrem I, Ruland C, Rumpsfeld M, Gammon D (2018). A person-centered integrated care quality framework, based on a qualitative study of patients' evaluation of care in light of chronic care ideals. BMC Health Serv Res.

[42] Coulter A, Roberts S, Dixon A (2013). The Kings Fund.

[43] Taylor C (1998). The Malaise Of Modernity.

[44] Turner G, Clegg A, British Geriatrics Society, Age UK, Royal College of General Practioners (2014). Best practice guidelines for the management of frailty: a British Geriatrics Society, Age UK and Royal College of General Practitioners report. Age Ageing.

[45] McBain H, Shipley M, Newman S (2015). The impact of self-monitoring in chronic illness on healthcare utilisation: a systematic review of reviews. BMC Health Serv Res.

[46] Ferdous R, Khan F, Sadiq R, Amyotte P, Veitch B (2013). Analyzing system safety and risks under uncertainty using a bow-tie diagram: an innovative approach. Process Saf Environ Prot.

[47] Bakker FC, Robben SH, Olde Rikkert MG (2011). Effects of hospital-wide interventions to improve care for frail older inpatients: a systematic review. BMJ Qual Saf.

[48] Eklund K, Wilhelmson K (2009). Outcomes of coordinated and integrated interventions targeting frail elderly people: a systematic review of randomised controlled trials. Health Soc Care Community.

[49] Vegesna A, Tran M, Angelaccio M, Arcona S (2017). Remote patient monitoring via non-invasive digital technologies: a systematic review. Telemed J E Health.

[50] Gammon D, Berntsen GK, Koricho AT, Sygna K, Ruland C (2015). The chronic care model and technological research and innovation: a scoping review at the crossroads. J Med Internet Res.

[51] Arksey H, O'Malley L (2005). Scoping studies: towards a methodological framework. Int J Soc Res Methodol.

[52] Levac D, Colquhoun H, O'Brien KK (2010). Scoping studies: advancing the methodology. Implement Sci.

[53] Higgins JPT, Green S (2011). Cochrane Handbook for Systematic Reviews of Interventions Version 6.

[54] Xue QL (2011). The frailty syndrome: definition and natural history. Clin Geriatr Med.

[55] Bleijenberg N, Drubbel I, Schuurmans MJ, Dam HT, Zuithoff NP, Numans ME, de Wit NJ (2016). Effectiveness of a proactive primary care program on preserving daily functioning of older people: a cluster randomized controlled trial. J Am Geriatr Soc.

[56] Blom J, den Elzen W, van Houwelingen AH, Heijmans M, Stijnen T, Van den Hout W, Gussekloo J (2016). Effectiveness and cost-effectiveness of a proactive, goal-oriented, integrated care model in general practice for older people. A cluster randomised controlled trial: Integrated Systematic Care for older People--the ISCOPE study. Age Ageing.

[57] Boult C, Leff B, Boyd CM, Wolff JL, Marsteller JA, Frick KD, Wegener S, Reider L, Frey K, Mroz TM, Karm L, Scharfstein DO (2013). A matched-pair cluster-randomized trial of guided care for high-risk older patients. J Gen Intern Med.

[58] Boult C, Reider L, Leff B, Frick KD, Boyd CM, Wolff JL, Frey K, Karm L, Wegener ST, Mroz T, Scharfstein DO (2011). The effect of guided care teams on the use of health services: results from a cluster-randomized controlled trial. Arch Intern Med.

[59] Boyd CM, Boult C, Shadmi E, Leff B, Brager R, Dunbar L, Wolff JL, Wegener S (2007). Guided care for multimorbid older adults. Gerontologist.

[60] Boyd CM, Shadmi E, Conwell LJ, Griswold M, Leff B, Brager R, Sylvia M, Boult C (2008). A pilot test of the effect of guided care on the quality of primary care experiences for multimorbid older adults. J Gen Intern Med.

[61] Council LS, Geffken D, Valeras AB, Orzano AJ, Rechisky A, Anderson S (2012). A medical home: changing the way patients and teams relate through patient-centered care plans. Fam Syst Health.

[62] Liss DT, Fishman PA, Rutter CM, Grembowski D, Ross TR, Johnson EA, Reid RJ (2013). Outcomes among chronically ill adults in a medical home prototype. Am J Manag Care.

[63] Martin CM, Vogel C, Grady D, Zarabzadeh A, Hederman L, Kellett J, Smith K, O'Shea B (2012). Implementation of complex adaptive chronic care: the Patient Journey Record system (PaJR). J Eval Clin Pract.

[64] Nelson KM, Helfrich C, Sun H, Hebert PL, Liu C, Dolan E, Taylor L, Wong E, Maynard C, Hernandez SE, Sanders W, Randall I, Curtis I, Schectman G, Stark R, Fihn SD (2014). Implementation of the patient-centered medical home in the Veterans Health Administration: associations with patient satisfaction, quality of care, staff burnout, and hospital and emergency department use. JAMA Intern Med.

[65] Ritchie J, Spencer L (2002). Qualitative data analysis for applied policy research. The Qualitative Researcher's Companion.

[66] Tjora A (2012). Qualitative research methods - a practical guide [Kvalitative forskningsmetoder i praksis.].

[67] Bleijenberg N, Ten Dam VH, Drubbel I, Numans ME, de Wit NJ, Schuurmans MJ (2016). Treatment fidelity of an evidence-based nurse-led intervention in a proactive primary care program for older people. Worldviews Evid Based Nurs.

[68] Foret GJ, Tanner E, Frey K, Reider L, Boult C (2009). Expanding the gerontological nursing role in Guided Care. Geriatr Nurs.

[69] Kearney LK, Post EP, Zeiss A, Goldstein MG, Dundon M (2011). The role of mental and behavioral health in the application of the patient-centered medical home in the Department of Veterans Affairs. Transl Behav Med.

[70] Reid RJ, Fishman PA, Yu O, Ross TR, Tufano JT, Soman MP, Larson EB (2009). Patient-centered medical home demonstration: a prospective, quasi-experimental, before and after evaluation. Am J Manag Care.

[71] Bertalanffy Lv (1950). An outline of general system theory. Br J Philos Sci.

[72] Weinelt B World Economic Forum White Paper.

[73] Stacey R (1995). The science of complexity: an alternative perspective for strategic change processes. Strat Mgmt J.

[74] (2012). Quality Management Principles.

[75] Deming W (2000). Out of the Crisis.

[76] Greenhalgh T, Papoutsi C (2018). Studying complexity in health services research: desperately seeking an overdue paradigm shift. BMC Med.

