# Supplementary material for: The Evidence Base for an Ideal Care Pathway for Frail Multimorbid Elderly: Combined Scoping and Systematic Intervention Review
Source: J Med Internet Res. 2019 Apr 22;21(4):e12517. doi: 10.2196/12517 (PMC6658285; doi:10.2196/12517)
Supplement: Multimedia Appendix 1 [file jmir_v21i4e12517_app1.docx]

## Appendix A

# More detail on Methods, and literature review search strings

## Details on paper inclusion and data extraction

We did consider registering the review in Prospero, but since this is in part a scoping review, this review was not eligible for registration. A research librarian in cooperation with members of the project group developed a search strategy. Individual searches were built and performed in each of the databases. Ten randomly chosen publications initially excluded in the “title and abstract” review were added to the full-text review, to ensure agreement in evaluation between the first and second review round. We did not include any of the initially excluded papers in the full-text review. The search is illustrated in figure X below.

At least three authors read each of the included studies and produced. The author group discussed each study to arrive at a final consensual textual description of each area and a summary for all seven studies on each of the Person-centered, Integrated, and Proactive-process elements and their digital support. The outline was read and discussed by all authors for reliability and validity across readers.

### Maturity scoring:

In an early version of the maturity matrix, we devised a maturity scoring system. In the final model, the maturity matrix, which was not used for scoring, we have mapped each key component to the steps in the patient pathway and is provided in the main paper.

However, since we used the earlier model for scoring, we present the earlier model here, together with scoring results. This adds the advantage of showing the development between models.

Appendix A, table 1: Care delivery of a Person-centered, Integrated, Proactive care process. Each cell represents a break-down element that is important for a mature and fully functional PIP-domain. The more components present, the higher the level of "Maturity of PCC model." Questions are to be seen as "guides" to understand the concept in each cell, not as scoring elements.

| **Care delivery** | | |  |
| --- | --- | --- | --- |
| **PCC** | **Integrated approach** | **Proactive approach** |  |
| No mention of person-centeredness | Diagnosis specific Evidence-Based Medicine | General self-management courses.  No risk management.  Low-threshold access to GP |  |
| **PCC is an undefined ideal:**  *Does the intervention use words like person-centered, patient-centered, client-centered?* | **Disease management across providers:**  Are there pre-defined standardized disease-specific care pathways across providers? | **Patient involvement and engagement**  Are patients supported in learning self-management skills for everyday care?  Can patients request health coaching to improve self-efficacy and behavioral change?  Do patients know what to do in the event of possible emergencies*?* |  |
| **PCC is understanding “what matters”**:  Is care sensitive to patient needs, values, preferences or “what matters to the person”?  Is patient involvement and engagement encouraged?  Is PCC operationalized by use of a method (i.e., motivational interview)? | **One person - one care plan:**  Is there a "role" (i.e., case-manager or team) who is responsible for a personal care plan development and follow-up?  Does the care plan cover all relevant health issues for the person? | **Self-managed early detection of decline**:  Have the most likely scenarios for undesired events been systematically identified?  Are patients (or someone close to the patient, family, home nurse, etc.) trained to detect early signals of decline?  Does the patient have a clear action plan in the case of alarming changes?  Does the patient have a low-threshold point of contact in case of questions or need for help to manage signals of decline? |  |
| **The care plan reflects "what matters."**  Is "what matters" translated into relevant, personalized goals for care?  Does the care plan reflect personalized goals?  Is there a planned evaluation of goal attainment together with the patient? | **From care plan to care delivery:**  Is there a system of resource booking linked to care plan activities?  In the case of multiple providers, are there coordination points (i.e., common meeting places, shared notes, etc) in place? | **System support for early detection**:  Is there a digital remote follow-up (i.e., sensors, etc.), or a professional follow-up, which detects early signals of decline?  If an alarm is triggered - is it clear who in the care system gets the alert? |  |
|  | **Monitoring of care delivery**  Is the care plan delivery monitored?  In the case of failure to deliver critical care according to plan, will a professional be alerted and take action? | **Alarms response and treatment:**  If an early detection triggers an alarm - is there a plan for a professional response?  Does the patient have access to an alarm kit (i.e. medication) for early treatment?  In case of an alarm which needs on site management - are there mobile resources who can reach the person’s location? |  |
|  |  | **Population risk stratification:**  Is available health data used for a population-health risk stratification?  If yes, is the high-risk group routed to a risk management process? |  |

Table 3: Relevant digital support components for a Person-centered, Integrated, Proactive care process. Each cell represents a break-down element that is important for a mature and fully digitalized PIP-domain. The more components present, the higher the level of PIP-digitalization. Questions are to be seen as “guides” to understand the concept in each cell, not as individual scoring elements.

| **Digital support** | | | |
| --- | --- | --- | --- |
| **PCC** | **Integrated care** | **Proactive care** |  |
| **None** | **Siloed Electronic Health Record** (EHR) | Standalone health and wellbeing applications  General health information |  |
| **Access to my health information:**  Does the patient have digital access to their electronic health record (EHR), care plan, referrals or lab results, in a format that lay people can understand? | **Care planning support:**  Does the system provide clinical decision support?  Are there digitized evidence based care pathways which outline care across providers for specific diseases?  Are there digital tools to build a personalized plan across conditions and providers? | **Digitally supported self-management:**  Are patients encouraged and trained to use digital self-management tools:   - to improve health literacy? - to set self-management goals and monitor progress? - To access decision support ? |  |
| **Support in deciding “What matters”?**  Does the patient have access to tools that support the formulation of "What matters"?  Can patients and clinicians review shared decision making tools together.  Can the patient share his/ her preferences for involvement and engagement with providers? | **Sharing the care plan**  Is the personal care plan digitally shared:   - across providers and organizations? - with the patient?   Is the care plan interactively updated across actors?  Is there a shared emergency/ crisis care plan? | **Digital early risk detection:**  Does the patient have digital sensors that monitor biologically relevant risk data?  Are there digital tools that support everyday functioning to reduce risk (i.e., pill reminders, fire detectors, GPS-sensors, fall detectors, video visits on demand, etc)?  Does the monitoring system send trigger alarms or alerts to relevant personnel or to the patient? |  |
| **Sharing: "What matters"**  Is "what matters" shared across care providers?  Are negotiated personalized goals for care shared across providers? | **The virtual care team:**  Does the digital system identify the multi-professional team, including the patient?  Is there support for sharing text, sound or pictures, (synchronously or asynchronously), across the team? | **Early intervention:**  If there is an early risk alert, is there digital decision support available to manage the risk situation? |  |
| **Patient feedback:**  Can patients provide digital feedback to providers?  Can the evaluation of personalized goals be reported digitally by the patient?  Is there a digital reporting of relevant patient-reported outcomes (PROMs). | **From care plan to delivery:**  Is there digital support for booking of resources to the care plan?  Do managers have access to work-flow optimization tools? | **Population risk stratification**  Does the care provider use health data for population risk stratification?  Are the population health outcomes (mortality, emergency admissions, etc.) monitored in digital outcomes registries? |  |
|  | **Monitoring of care delivery**  Is there digital monitoring of care delivery according to plan?  Will a professional be notified in case of critical deviations in the delivery of the care plan? |  |  |

We awarded one point for each key component that was present in the intervention. Total maturity score is presented as a % of scored points, by maximum possible points, within each active ingredient area. Pairs of authors independently mapped and scored intervention maturity. In cases of disagreements, the authors discussed the study until they reached consensus. We present results as maturity spider diagrams.

Terminology in this multi-disciplinary field is challenging, even to a group of closely collaborating health and e-health experts. However, the Digi PCC, Integrated and Proactive-reflection model did provide a starting point for fruitful discussions which helped us build a shared mental model of the overarching idealized iPP, and their components involved in PCC, Integrated and Proactive care. We underline that the model at this stage is useful for reflection and dialogue, but does not have the validity and reliability of a measurement instrument.

*Appendix A - Figure 4: Spider diagrams of Maturity of Digi-PIP care in 7 included studies. Each axis is presented as a % of achieved points by maximum possible points. The 3 Care elements (top half) were Person-centered (Max: 3 points) Integrated: (max 4 points) and Proactive care (max 5 points). The three Digital axes (bottom half) were digital person-centered (Max: 4 points) digital Integrated: (max 5 points) and digital proactive care (max 4 points).*


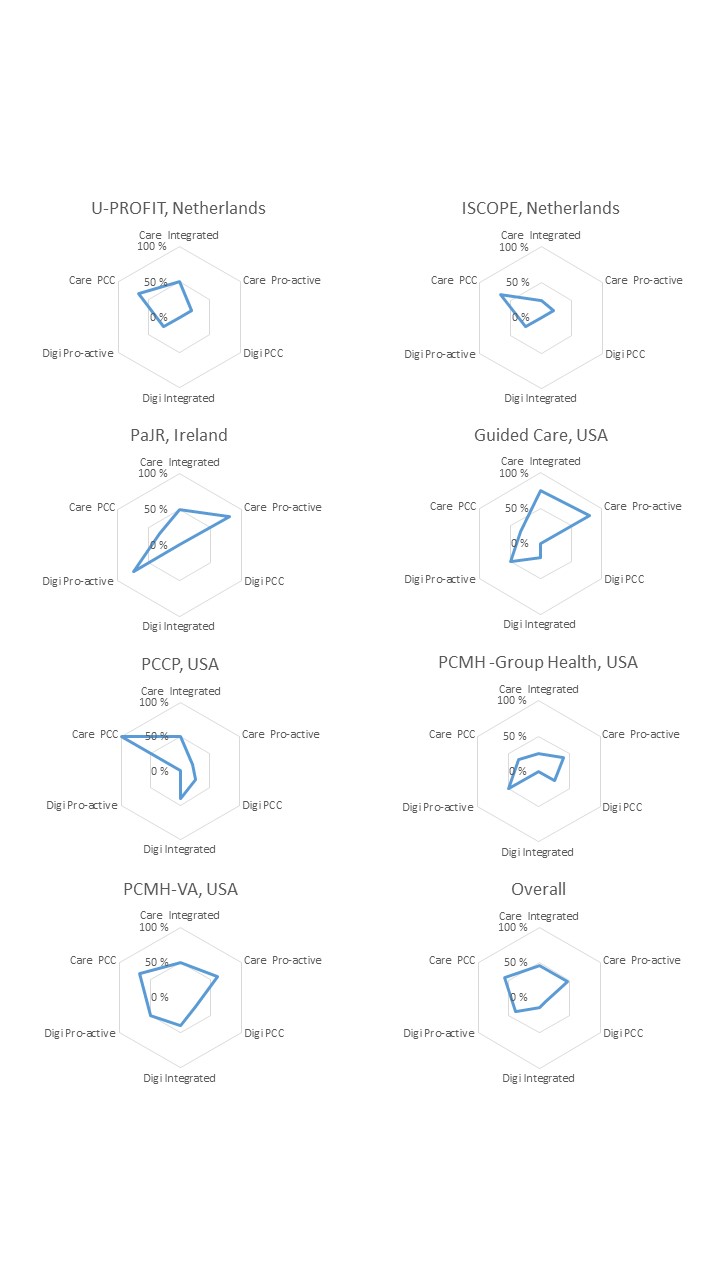


U-PROFIT - Utrecht PROactive Frailty Intervention Trial, ISCOPE - Integrated Systematic Care for Older People, PCMH-VA - Person-Centered Medical Home- Veterans Health Administration.

## Quality of study evaluation

We present the design and quality evaluations of the effect evaluation studies in Appendix A, Table 2. The three cluster randomized trials (U-PROFIT, ISCOPE and Guided Care) [55-57] hold high methodological quality. The PCMH-VA and GH studies based on registry data did not satisfactorily account for case-mix, as they showed important differences at baseline, which were not adjusted for in all their analyses [62,64]. The two smaller trials, PCCP and PaJR [61,63] were more exploratory, with a focus on describing an intervention philosophy and tying these to mechanisms for action. The studies excluded from the effect evaluation also included measures on employee satisfaction and burnout.

Appendix A, Table 2: Study design and quality for included studies ad modum de Bruin[16] (0-not satisfactory, 1-satisfactory, NA-not applicable).

| Paper | Design | Randomized? | Equal Baseline? | Compliance | Dropout rates | Intention to treat | Adjusted | # possible* | # fulfilled* |
| --- | --- | --- | --- | --- | --- | --- | --- | --- | --- |
| U-PROFIT [55] | Cluster randomization at general practices level. Single blinded. | 1 | 1 | 0 | 1 | 1 | 1 | 6 | 5 |
| ISCOPE [56] | Single-blinded cluster randomized controlled trial at the general practice level. | 1 | 1 | 1 | 1 | 1 | 1 | 6 | 6 |
| PaJR [63] | Community-based cohort study – random assignment at patient level to intervention and usual care group, with outcome evaluation. | 1 | 1 | 0 | 0 | 0 | 0 | 6 | 2 |
| Guided Care [57] | A single blind, cluster-randomized controlled trial of Guided Care. Randomized at "pod" level. (pod=GP and his/her patients). | 1 | 1 | 0 | 1 | 1 | 1 | 6 | 5 |
| PCCP [61] | A qualitative in-depth interview with patients and health professionals. Before-After comparisons. | NA | NA | NA | NA | NA | NA | 0 | 0 |
| PCMH-VA [64] | Clinic-level observational analysis using aggregate patient and employee survey data, and administrative data of clinics with a high and low implementation of PCMH. | 0 | NA | 1 | 1 | NA | 1 | 4 | 3 |
| PCMH-GH [62] | Non-equivalent controlled before-after design. Based on registry data at patient level from 1 intervention clinic compared to 20 non-intervention clinics. | 0 | 0 | 0 | 1 | 1 | 0 | 6 | 2 |

# Possible: Possible maximum score, # fulfilled: Number of criteria satisfactorily met. GP- General Practitioner. U-PROFIT - Utrecht PROactive Frailty Intervention Trial, ISCOPE - Integrated Systematic Care for Older People, PCMH-VA - Person-Centered Medical Home- Veterans Health Administration.

## Appendix B

### *Ovid MEDLINE(R) Epub Ahead of Print, In-Process & Other*

### *Non-Indexed Citations, Ovid MEDLINE(R) Daily and Ovid MEDLINE(R)*

|  | exp Patient-centered care/ |
| --- | --- |
|  | (patient-cent* or person-cent* or people-cent* or client-cent*).ti,ab. |
|  | (patient-oriented or person-oriented or client-oriented).ti,ab. |
|  | (patient-focused or person-focused or client-focused).ti,ab. |
|  | Patient Participation/ |
|  | patient participation.ti,ab. |
|  | Patient care planning/ |
|  | (care planning or healthcare planning or health care planning).ti,ab. |
|  | Decision making/ |
|  | (decision making).ti,ab. |
|  | (user-cent* or consumer-cent*).ti,ab. |
|  | Self-care/ |
|  | (self-care or self-management).ti,ab. |
|  | ((goal-oriented ADJ3 care) or (goal-oriented ADJ2 healthcare)).ti,ab. |
|  | Or/1-14 |
|  | Ambulatory care/ |
|  | ((ambulatory ADJ3 care) or (ambulatory ADJ2 healthcare)).ti,ab. |
|  | (amenable mortalit* or amenable morbidit*).ti,ab. |
|  | integrated health* ADJ3 service*.ti,ab. |
|  | proactive health* management.ti,ab. |
|  | ((planned ADJ3 care) or (planned ADJ2 healthcare)).ti,ab. |
|  | ((outpatient ADJ2 care) or (outpatient healthcare) or (outpatient ADJ2 service)).ti,ab. |
|  | Or/16-22 |
|  | Delivery of health care/ |
|  | Delivery of health care, Integrated/ |
|  | (integrated care pathway* or integrated healthcare pathway*).ti,ab. |
|  | Integrated ADJ3 pathway.ti,ab |
|  | integrated ADJ2 (care or healthcare or service*).ti,ab |
|  | (continuous or shared) ADJ2 (care or healthcare).ti,ab. |
|  | co-production of health.ti,ab. |
|  | (intersectoral action or vertical ADJ2 program*).ti,ab. |
|  | whole-system thinking.ti,ab. |
|  | ((collaborat* ADJ3 (care or healthcare)) or (collaborat* ADJ2 (management or health care coordination))).ti,ab. |
|  | ((coordinat* ADJ3 (care or healthcare)) or (co-ordinated ADJ3 (care or healthcare)) or coordinated program* or co-ordinated program*).ti,ab. |
|  | exp continuity of patient care/ |
|  | continuity ADJ2 (care or healthcare).ti,ab. |
|  | empowerment or engagement.ti,ab. |
|  | exp patient care planning/ |
|  | (patient ADJ2 (care or healthcare) ADJ1 planning).ti,ab. |
|  | comprehensive health care/ |
|  | comprehensive ADJ3 (care or healthcare).ti,ab. |
|  | Case management/ |
|  | “case management”.ti,ab. |
|  | “Chronic care model”.ti,ab. |
|  | Or/24-44 |
|  | 15 AND 23 AND 45 |
|  | exp Aged/ |
|  | Aged, 80 and over/ |
|  | Frail elderly/ |
|  | (Elder* or aged).ti,ab. |
|  | > 65.ti,ab. |
|  | (chronic or complex).ti,ab. |
|  | (long term ADJ2 care).ti,ab. |
|  | functionally impaired elder*.ti,ab. |
|  | frail older adult*.ti,ab. |
|  | Or/47-55 |
|  | 46 AND 56 |

## Web of Science

### *Web of Science Core Collection*

|  |  |
| --- | --- |
|  | TS= (patient-cent* OR person-cent* OR people-cent* OR client-cent*) |
|  | TS= (patient-oriented OR person-oriented OR client-oriented) |
|  | TS= (patient-focused OR person-focused OR client-focused) |
|  | TS= “patient participation” |
|  | TS= “care planning” |
|  | TS= “decision making” (include both decision-making and decision making) |
|  | TS= (user-cent* OR consumer-cent*) |
|  | TS=(”self-care”OR “self-management”) |
|  | TS= (goal-oriented NEAR/2 (care OR healthcare)) |
|  | **OR/1-9** |
|  | TS=(ambulatory NEAR/2 (care OR healthcare)) |
|  | TS= “supported self-care” |
|  | TS=(“ambenable mortalit*” OR “amenable morbidit*”) |
|  | TS=“proactive health* management” |
|  | TS=(planned NEAR/2 (care OR healthcare)) |
|  | TS=(outpatient NEAR/1 (care OR healthcare) OR (outpatient NEAR/1 service)) |
|  | **OR/11-16** |
|  | TS= (integrated NEAR/1 (care OR healthcare)) |
|  | TS= “integrated pathway” |
|  | TS=(integrated NEAR/1 care NEAR/1 pathway) |
|  | TS=(integrated NEAR/1 healthcare NEAR/1 pathway) |
|  | TS=“integrated health* service*” |
|  | TS= (continuous NEAR/1 (care OR healthcare) OR shared NEAR/1 (care OR healthcare)) |
|  | TS=”co-production of health*” |
|  | TS= (“intersectoral action” OR “vertical NEAR/1 program*”) |
|  | TS= “whole-system thinking” |
|  | TS=(collaborative NEAR/1 (care OR healthcare)) |
|  | TS=(health care NEAR/1 (coordination OR co-ordination) OR healthcare NEAR/1(coordination OR co-ordination) OR care NEAR/1 (coordination OR co-ordination)) |
|  | TS=(continuity NEAR/2 care) |
|  | TS= (empowerment OR engagement) |
|  | TS= (patient NEAR/1 (care OR healthcare) NEAR/1 planning) |
|  | TS= (comprehensive NEAR/1 (care or healthcare)) |
|  | TS =“case management” |
|  | TS=”chronic care model” |
|  | **OR/18-34** |
|  | **10 AND 17 AND 35** |
|  | TS= (Elder* OR aged OR “>65” OR chronic OR complex OR “frail older adult*” OR “functionally impaired elder*” ) |
|  | TS= (long term NEAR/1 (care OR healthcare)) |
|  | **Or/37-38** |
|  | **36 AND 39** |

## Scopus

### *Searched in title and abstract (TITLE-ABS)*

|  | patient-cent* OR person-cent* OR people-cent* OR client-cent* |
| --- | --- |
|  | patient-oriented OR person-oriented OR client-oriented |
|  | patient-focused OR person-focused OR client-focused |
|  | “patient participation” |
|  | “*care planning” |
|  | “decision making” |
|  | user-cent* OR consumer-cent* |
|  | self-care OR self-management |
|  | goal-oriented PRE/2 *care |
|  | **OR/1-9** |
|  | “ambulatory *care” |
|  | “ambenable mortalit*” OR “amenable morbidit*” |
|  | “proactive health* management” |
|  | “planned PRE/2 *care” |
|  | (outpatient PRE/1 *care) OR (outpatient PRE/1 service) |
|  | **OR/11-16** |
|  | integrated PRE/1 *care |
|  | “integrated pathway” OR “integrated patient pathway” OR “integrated *care pathway” OR “integrated health* service*” |
|  | continuous PRE/1 *care |
|  | shared PRE/1 *care |
|  | “co-production of health*” |
|  | “intersectoral action” OR vertical PRE/1 program* |
|  | “whole-system thinking” |
|  | ((Collaborat* PRE/2 *care) |
|  | (Collaborat* PRE/2 “*care coordination”) OR (Collaborat* PRE/2 “*care co-ordination”) OR (Collaborat* PRE/2 management) |
|  | **OR/#17-26 (Scopus can not manage broad combinations)** |
|  | (Healthcare OR “health care” OR care) PRE/1 (coordinat* or co-ordinat*)) |
|  | (“coordinated program*” OR “co-ordinated program*”) |
|  | continuity W/2 *care |
|  | empowerment OR engagement |
|  | “patient *care planning” |
|  | comprehensive PRE/2 *care |
|  | “case management” |
|  | “chronic care model” |
|  | **OR/27-32** |
|  | **OR/33-34** |
|  | **OR/26,35,36** |
|  | **10 AND 17 AND 30** |
|  | “frail elder*” |
|  | Elder* OR aged. |
|  | > 65 |
|  | chronic OR complex |
|  | “long term care” |
|  | “functionally impaired elder*” |
|  | “frail older adult*” |
|  | **OR/39-45** |
|  | **38 AND 46** |

# REFERENCES

1. Afshar S, Roderick PJ, Kowal P, Dimitrov BD, Hill AG. Global Patterns of Multimorbidity: A Comparison of 28 Countries Using the World Health Surveys. In: Hoque MN, Pecotte B, McGehee MA, eds. Applied Demography and Public Health in the 21st Century. Cham: Springer International Publishing; 2017:381-402, Doi: 10.1007/978-3-319-43688-3_21.

2. Violan C, Foguet-Boreu Q, Flores-Mateo G, et al. Prevalence, determinants and patterns of multimorbidity in primary care: a systematic review of observational studies. PLoS One*.* 2014; (Vol 9) 7:e102149. PMID: 25048354.

3. van Oostrom SH, Gijsen R, Stirbu I, et al. Time trends in prevalence of chronic diseases and multimorbidity not only due to aging: data from general practices and health surveys. PLoS One*.* 2016; (Vol 11) 8:e0160264. PMID: 27482903.

4. Wang L, Si L, Cocker F, Palmer AJ, Sanderson K. A Systematic Review of Cost-of-Illness Studies of Multimorbidity. Applied health economics and health policy*.* 2017:1-15. PMID: 28856585.

5. Zulman DM, Pal Chee C, Wagner TH, et al. Multimorbidity and healthcare utilisation among high-cost patients in the US Veterans Affairs Health Care System. BMJ Open*.* 2015; (Vol 5) 4. PMID: 25882486.

6. Heiberg I. High utilisation patients in somatic specialist healthcare in Northern Norway. [Storforbrukere av somatisk spesialisthelsetjeneste i Helse Nord]*.* SKDE;2015. ISBN : 978-82-93141-18-1.

7. Ossebaard HC, Van Gemert-Pijnen L. eHealth and quality in health care: implementation time. International Journal for Quality in Health Care*.* 2016; (Vol 28) 3:415-419. PMID: 27029590.

8. Conway ME. How do committees invent? Datamation*.* 1968; (Vol 14) 4:28-31.

9. Coiera E. Four rules for the reinvention of health care. BMJ*.* 2004; (Vol 328) 7449:1197-1199. PMID: 15142933.

10. Berwick DM, Nolan TW, Whittington J. The Triple Aim: Care, Health, And Cost. Health Affairs*.* 2008; (Vol 27) 3:759-769. PMID: 18474969.

11. PCPCC Map Tools. (2015). Patient-Centered Medical Home 201: A Snapshot of the Evidence. 2015: Archived at: http://www.webcitation.org/73DIUAtHF.

12. WHO. Framework on integrated, people-centred health services. Report by the Secretariat. 2016: Archived at: http://www.webcitation.org/73DWSHo7t.

13. Rich E LD, J L, M. P. Coordinating Care for Adults With Complex Care Needs in the Patient-Centered Medical Home: Challenges and Solutions. White Paper 2012: Archived at: http://www.webcitation.org/73DWky5eg.

14. Joint_Action_CHRODIS. Report on care pathways approaches for multimorbid chronic patients. CHRODIS - Addressing chronic disease and healthy ageing across the life cycle, 2017: Archived at: http://www.webcitation.org/73DWtuDxj.

15. Coulter A, Entwistle Vikki A, Eccles A, Ryan S, Shepperd S, Perera R. Personalised care planning for adults with chronic or long-term health conditions. Cochrane Database of Systematic Reviews*.* 2015; 3. PMID: 25733495.

16. de Bruin SR, Versnel N, Lemmens LC, et al. Comprehensive care programs for patients with multiple chronic conditions: A systematic literature review. Health Policy*.* 2012; (Vol 107) 2-3:108-145. PMID: 22884086.

17. Steventon A, Bardsley M, Billings J, et al. Effect of telehealth on use of secondary care and mortality: findings from the Whole System Demonstrator cluster randomised trial. BMJ: British Medical Journal*.* 2012; (Vol 344). PMID: 22723612.

18. Hallberg IR, Kristensson J. Preventive home care of frail older people: a review of recent case management studies. Journal of Clinical Nursing*.* 2004; (Vol 13) 6B:112-120. PMID: 15724826.

19. Ekeland AG, Bowes A, Flottorp S. Effectiveness of telemedicine: a systematic review of reviews. International Journal of Medical Informatics*.* 2010; (Vol 79) 11:736-771. PMID: 20884286.

20. Hersh WR, Totten AM, Eden KB, et al. Outcomes From Health Information Exchange: Systematic Review and Future Research Needs. JMIR Medical Informatics*.* 2015; (Vol 3) 4:e39. PMID: 26678413.

21. Woods SS, Evans NC, Frisbee KL. Integrating patient voices into health information for self-care and patient-clinician partnerships: Veterans Affairs design recommendations for patient-generated data applications. Journal of the American Medical Informatics Association*.* 2016; (Vol 23) 3:491-495. PMID: 26911810.

22. Ham C, York N, Sutch S, Shaw R. Hospital bed utilisation in the NHS, Kaiser Permanente, and the US Medicare programme: analysis of routine data. BMJ*.* 2003; (Vol 327) 7426:1257-. PMID: 14644968.

23. Feachem RGA, Sekhri NK, White KL. Getting more for their dollar: a comparison of the NHS with California's Kaiser Permanente. BMJ*.* 2002; (Vol 324) 7330:135-141. PMID: 11799029.

24. Driscoll DL, Hiratsuka V, Johnston JM, et al. Process and Outcomes of Patient-Centered Medical Care With Alaska Native People at Southcentral Foundation. The Annals of Family Medicine*.* 2013; (Vol 11) Suppl 1:S41-S49. PMID: 23690385.

25. Rosland AM, Nelson K, Sun H, et al. The patient-centered medical home in the Veterans Health Administration. The American journal of managed care*.* 2013; (Vol 19) 7:e263-272. PMID: 23919446.

26. Busse R, Stahl J. Integrated Care Experiences And Outcomes In Germany, The Netherlands, And England. Health Affairs*.* 2014; (Vol 33) 9:1549-1558. PMID: 25201659.

27. Hughes G. New models of care: the policy discourse of integrated care. 2017.

28. Salisbury C, Man M-S, Bower P, et al. Management of multimorbidity using a patient-centred care model: a pragmatic cluster-randomised trial of the 3D approach. The Lancet*.* 2018; (Vol 392) 10141:41-50. PMID: 29961638.

29. Scott IA. Chronic disease management: a primer for physicians. Intern Med J*.* 2008; (Vol 38) 6:427-437. PMID: 18298554.

30. Beswick AD, Rees K, Dieppe P, et al. Complex interventions to improve physical function and maintain independent living in elderly people: a systematic review and meta-analysis. The Lancet*.* 2008; (Vol 371) 9614:725-735. PMID: 18313501.

31. Lemmens LC, Molema CCM, Versnel N, Baan CA, de Bruin SR. Integrated care programs for patients with psychological comorbidity: A systematic review and meta-analysis. Journal of Psychosomatic Research*.* 2015; (Vol 79) 6:580-594. PMID: 26354890.

32. Valentijn PP, Schepman SM, Opheij W, Bruijnzeels MA. Understanding integrated care: a comprehensive conceptual framework based on the integrative functions of primary care. International Journal of Integrated Care*.* 2013; (Vol 13) 1. PMID: 23687482.

33. Coulter A, Ellins J. Effectiveness of strategies for informing, educating, and involving patients. British Medical Journal*.* 2007; (Vol 335) 7609:24. PMID: 17615222 .

34. Stellefson M, Dipnarine K, Stopka C. The Chronic Care Model and Diabetes Management in US Primary Care Settings: A Systematic Review. Prev Chronic Dis*.* 2013; (Vol 10). PMID: 23428085.

35. Huntley AL, Thomas R, Mann M, et al. Is case management effective in reducing the risk of unplanned hospital admissions for older people? A systematic review and meta-analysis. Family practice*.* 2013; (Vol 30) 3:266-275. PMID: 23315222.

36. Tenforde AS, Hefner JE, Kodish-Wachs JE, Iaccarino MA, Paganoni S. Telehealth in Physical Medicine and Rehabilitation: A Narrative Review. Pm&R*.* 2017; (Vol 9) 5:S51-S58. PMID: 28527504.

37. Kaplan B, Harris-Salamone KD. Health IT Success and Failure: Recommendations from Literature and an AMIA Workshop. Journal of the American Medical Informatics Association*.* 2009; (Vol 16) 3:291-299. PMID: 19261935.

38. Greenhalgh T, Russell J. Why do evaluations of eHealth programs fail? An alternative set of guiding principles. PLoS Medicine*.* 2010; (Vol 7) 11:e1000360. PMID: 21072245.

39. Person-centered care made simple. 2014: Archived at: http://www.webcitation.org/73DX9aYtT

40. Ehrlich C, Kendall E, Muenchberger H, Armstrong K. Coordinated care: what does that really mean? Health Soc Care Community*.* 2009; (Vol 17) 6:619-627. PMID: 19469914.

41. Berntsen GR, Høyem A, Lettrem I, Ruland C, Rumpsfeld M, Gammon DB. A person-centered integrated care quality framework. A qualitative study of patients' evaluation of care in light of chronic care ideals. BMC Health services research 2018; 18. PMID: 29925357.

42. Coulter A, Roberts S, Dixon A. Delivering better services for people with long-term conditions. Building the house of care. 2013: Archived at: http://www.webcitation.org/73DY4ZvtB.

43. Taylor C. The Malaise of Modernity Toronto: House of Anansi Press; 1991, Doi: ISBN 0-88784-520-7.

44. Turner G, Clegg A. Best practice guidelines for the management of frailty: a British Geriatrics Society, Age UK and Royal College of General Practitioners report. Age and Ageing*.* 2014; (Vol 43) 6:744-747. PMID: 25336440.

45. McBain H, Shipley M, Newman S. The impact of self-monitoring in chronic illness on healthcare utilisation: a systematic review of reviews. Bmc Health Services Research*.* 2015; (Vol 15). PMID: 26684011.

46. Ferdous R, Khan F, Sadiq R, Amyotte P, Veitch B. Analyzing system safety and risks under uncertainty using a bow-tie diagram: An innovative approach. Process Safety and Environmental Protection*.* 2013; (Vol 91) 1:1-18.

47. Bakker FC, Robben SHM, Rikkert MGMO. Effects of hospital-wide interventions to improve care for frail older inpatients: a systematic review. BMJ Qual Saf*.* 2011; (Vol 20) 8:680-691. PMID: 21355019.

48. Eklund K, Wilhelmson K. Outcomes of coordinated and integrated interventions targeting frail elderly people: a systematic review of randomised controlled trials. Health Soc Care Community*.* 2009; (Vol 17) 5:447-458. PMID: 19245421.

49. Vegesna A, Tran M, Angelaccio M, Arcona S. Remote patient monitoring via non-invasive digital technologies: a systematic review. Telemedicine and e-Health*.* 2017; (Vol 23) 1:3-17. PMID: 27116181.

50. Gammon D; Berntsen GKR M, PhD; Koricho AT; Sygna K; Ruland CM. The Chronic Care Model and Technological Research & Innovation: A scoping review at the crossroads. A systematic review JMIR*.* 2014; (Vol 17) 2. PMID: 25677200.

51. Arksey H, O'Malley L. Scoping studies: towards a methodological framework. International journal of social research methodology*.* 2005; (Vol 8) 1:19-32.

52. Levac D, Colquhoun H, O'Brien KK. Scoping studies: advancing the methodology. Implementation Science*.* 2010; (Vol 5) 1:69. PMID: 20854677, Doi: 10.1186/1748-5908-5-69.

53. Cochrane Handbook for Systematic Reviews of Interventions Version 6. 2011: Archived at: ttp://www.webcitation.org/73DXqSxbS.

54. Xue Q-L. The frailty syndrome: definition and natural history. J Clinics in geriatric medicine*.* 2011; (Vol 27) 1:1-15. PMID: 21093718.

55. Bleijenberg N, Drubbel I, Schuurmans MJ, et al. Effectiveness of a Proactive Primary Care Program on Preserving Daily Functioning of Older People: A Cluster Randomized Controlled Trial. J Am Geriatr Soc*.* 2016; (Vol 64) 9:1779-1788. PMID: 27459236 .

56. Blom J, den Elzen W, van Houwelingen AH, et al. Effectiveness and cost-effectiveness of a proactive, goal-oriented, integrated care model in general practice for older people. A cluster randomised controlled trial: Integrated Systematic Care for older People-the ISCOPE study. Age and Ageing*.* 2016; (Vol 45) 1:30-41. PMID: 26764392.

57. Boult C, Leff B, Boyd CM, et al. A Matched-Pair Cluster-Randomized Trial of Guided Care for High-Risk Older Patients. Journal of General Internal Medicine*.* 2013; (Vol 28) 5:612-621. PMID: 23307395.

58. Boult C, Reider L, Leff B, et al. The Effect of Guided Care Teams on the Use of Health Services Results From a Cluster-Randomized Controlled Trial. Archives of Internal Medicine*.* 2011; (Vol 171) 5:460-466. PMID: 21403043.

59. Boyd CM, Boult C, Shadmi E, et al. Guided care for multimorbid older adults. Gerontologist*.* 2007; (Vol 47) 5:697-704. PMID: 17989412.

60. Boyd CM, Shadmi E, Conwell LJ, et al. A pilot test of the effect of guided care on the quality of primary care experiences for multimorbid older adults. Journal of General Internal Medicine*.* 2008; (Vol 23) 5:536-542. PMID: 18266045.

61. Council LS, Geffken D, Valeras AB, Orzano AJ, Rechisky A, Anderson S. A medical home: changing the way patients and teams relate through patient-centered care plans. Families, Systems, & Health*.* 2012; (Vol 30) 3:190. PMID: 22985385.

62. Liss DT, Fishman PA, Rutter CM, et al. Outcomes among chronically ill adults in a medical home prototype. American Journal of Managed Care*.* 2011; (Vol 19) 10:e348-358. PMID: 24304182.

63. Martin CM, Vogel C, Grady D, et al. Implementation of complex adaptive chronic care: the Patient Journey Record system (PaJR). Journal of Evaluation in Clinical Practice*.* 2012; (Vol 18) 6:1226-1234. PMID: 22816797.

64. Nelson KM, Helfrich C, Sun H, et al. Implementation of the patient-centered medical home in the Veterans Health Administration: associations with patient satisfaction, quality of care, staff burnout, and hospital and emergency department use. JAMA Internal Medicine*.* 2014; (Vol 174) 8:1350-1358. PMID: 25055197.

65. Ritchie J, Spencer L. Qualitative data analysis for applied policy research. In: The qualitative researcher's companion. Sage Los Angeles, CA; 2002:305-329.

66. Tjora A. [Qualitative research methods - a practical guide] Kvalitative forskningsmetoder i praksis 2nd edition ed. Oslo: Gyldendal norsk forlag AS; 2012.

67. Bleijenberg N, ten Dam VH, Drubbel I, Numans ME, de Wit NJ, Schuurmans MJ. Treatment Fidelity of an Evidence-Based Nurse-Led Intervention in a Proactive Primary Care Program for Older People. Worldviews on Evidence-Based Nursing*.* 2016; (Vol 13) 1:75-84. PMID: 26873373.

68. Giddens JF, Tanner E, Frey K, Reider L, Boult C. Expanding the gerontological nursing role in Guided Care. In: Elsevier; 2009. PMID: 19839117.

69. Kearney LK, Post EP, Zeiss A, Goldstein MG, Dundon M. The role of mental and behavioral health in the application of the patient-centered medical home in the Department of Veterans Affairs. Translational behavioral medicine*.* 2011; (Vol 1) 4:624-628. PMID: 24073086.

70. Reid RJ, Fishman PA, Yu O, et al. Patient-Centered Medical Home Demonstration: A Prospective, Quasi-Experimental, Before and After Evaluation. Am J Manag Care*.* 2009; (Vol 15) 9:e71-e87. PMID: 19728768.

71. Bertalanffy L. An outline of general system theory. The British Journal for the Philosophy of science*.* 1950; 2:134-165.

72. Weinelt B. World Economic Forum White Paper. Digital Transformation of Industries: In collaboration withAccenture. Healthcare Industry., 2016: Archived at: http://www.webcitation.org/73DJ2NLHO.

73. Stacey RD. The science of complexity: An alternative perspective for strategic change processes. Strategic management journal*.* 1995; (Vol 16) 6:477-495.

74. ISO9000. Quality management principles*.* 2012. ISBN 978-92-67-10573-4.

75. Deming WE. Out of the Crisis. Cambridge, Massachustetts, London, England: MIT Press. Kindle Edition; 2000, Doi: 0262541157.

76. Greenhalgh T, Papoutsi C. Studying complexity in health services research: desperately seeking an overdue paradigm shift. In: BioMed Central; 2018. PMID: 29921272.
