# Supplementary material for: The Evidence Base for an Ideal Care Pathway for Frail Multimorbid Elderly: Combined Scoping and Systematic Intervention Review
Source: J Med Internet Res. 2019 Apr 22;21(4):e12517. doi: 10.2196/12517 (PMC6658285; doi:10.2196/12517)
Supplement: Multimedia Appendix 2 [file jmir_v21i4e12517_app2.pptx]

## Slide 1
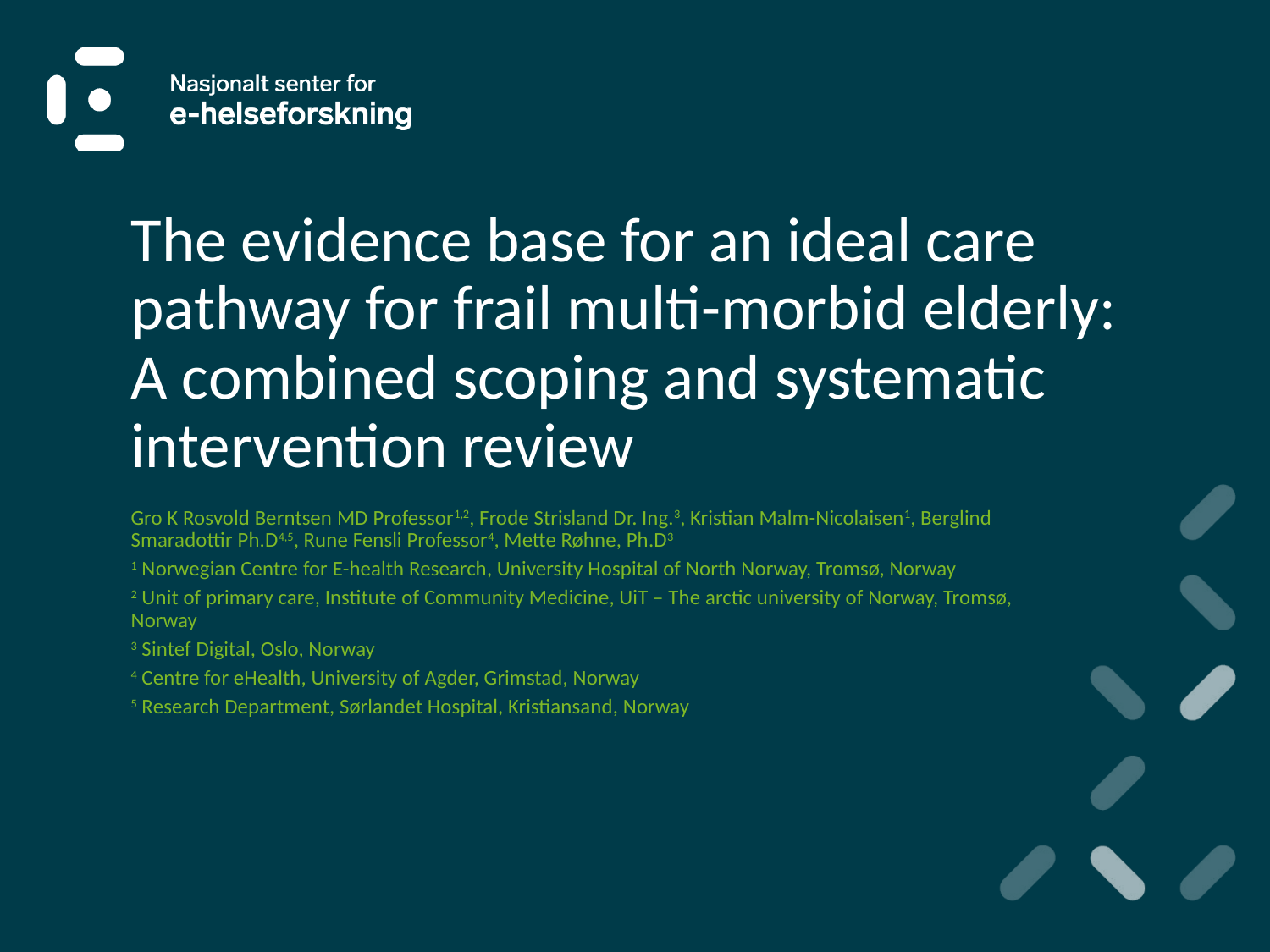

# The evidence base for an ideal care pathway for frail multi-morbid elderly: A combined scoping and systematic intervention review
Gro K Rosvold Berntsen MD Professor1,2, Frode Strisland Dr. Ing.3, Kristian Malm-Nicolaisen1, Berglind Smaradottir Ph.D4,5, Rune Fensli Professor4, Mette Røhne, Ph.D3
1 Norwegian Centre for E-health Research, University Hospital of North Norway, Tromsø, Norway
2 Unit of primary care, Institute of Community Medicine, UiT – The arctic university of Norway, Tromsø, Norway
3 Sintef Digital, Oslo, Norway
4 Centre for eHealth, University of Agder, Grimstad, Norway
5 Research Department, Sørlandet Hospital, Kristiansand, Norway

## Slide 2
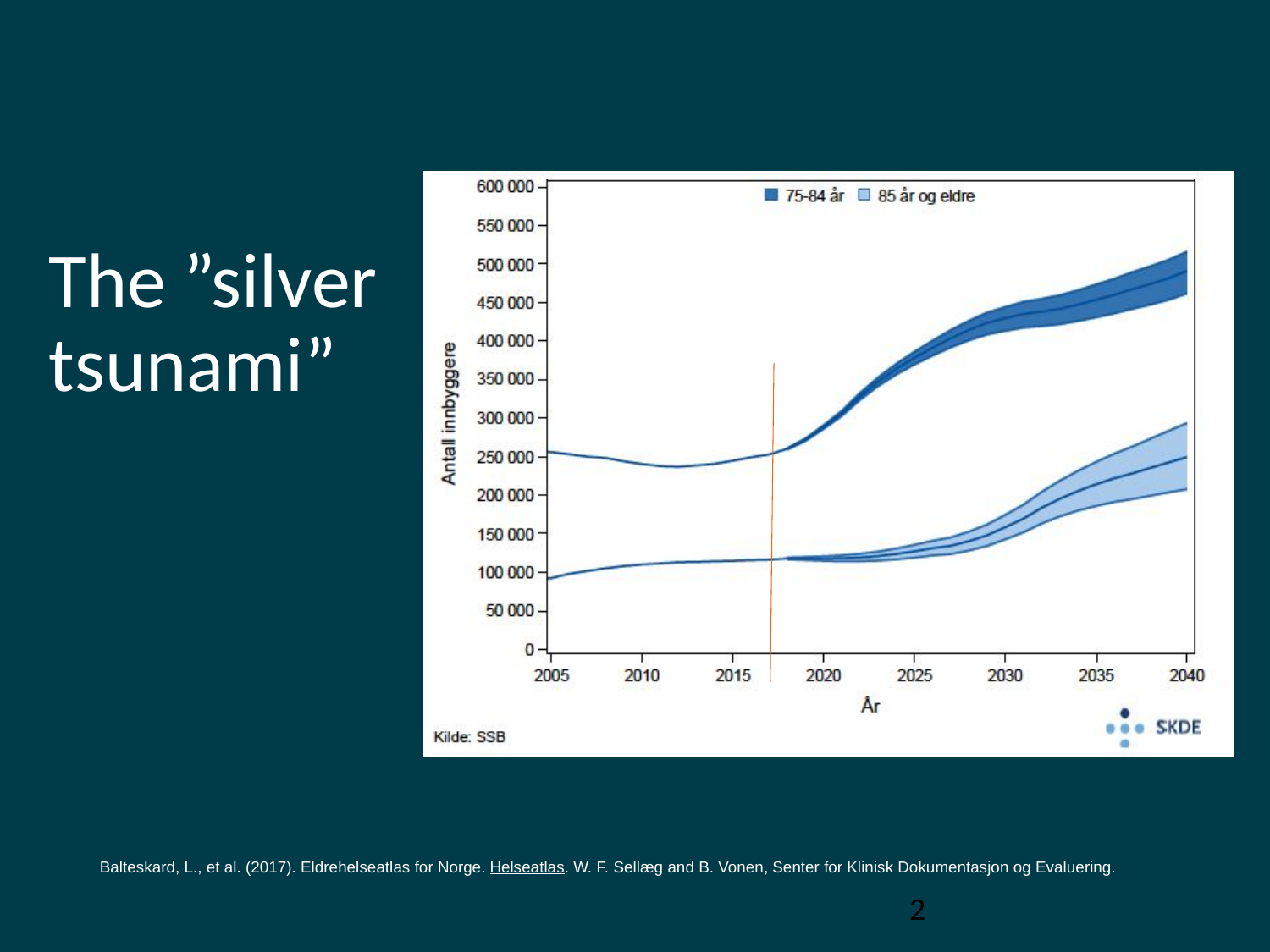

# The ”silver tsunami”
Balteskard, L., et al. (2017). Eldrehelseatlas for Norge. Helseatlas. W. F. Sellæg and B. Vonen, Senter for Klinisk Dokumentasjon og Evaluering.
2

## Slide 3
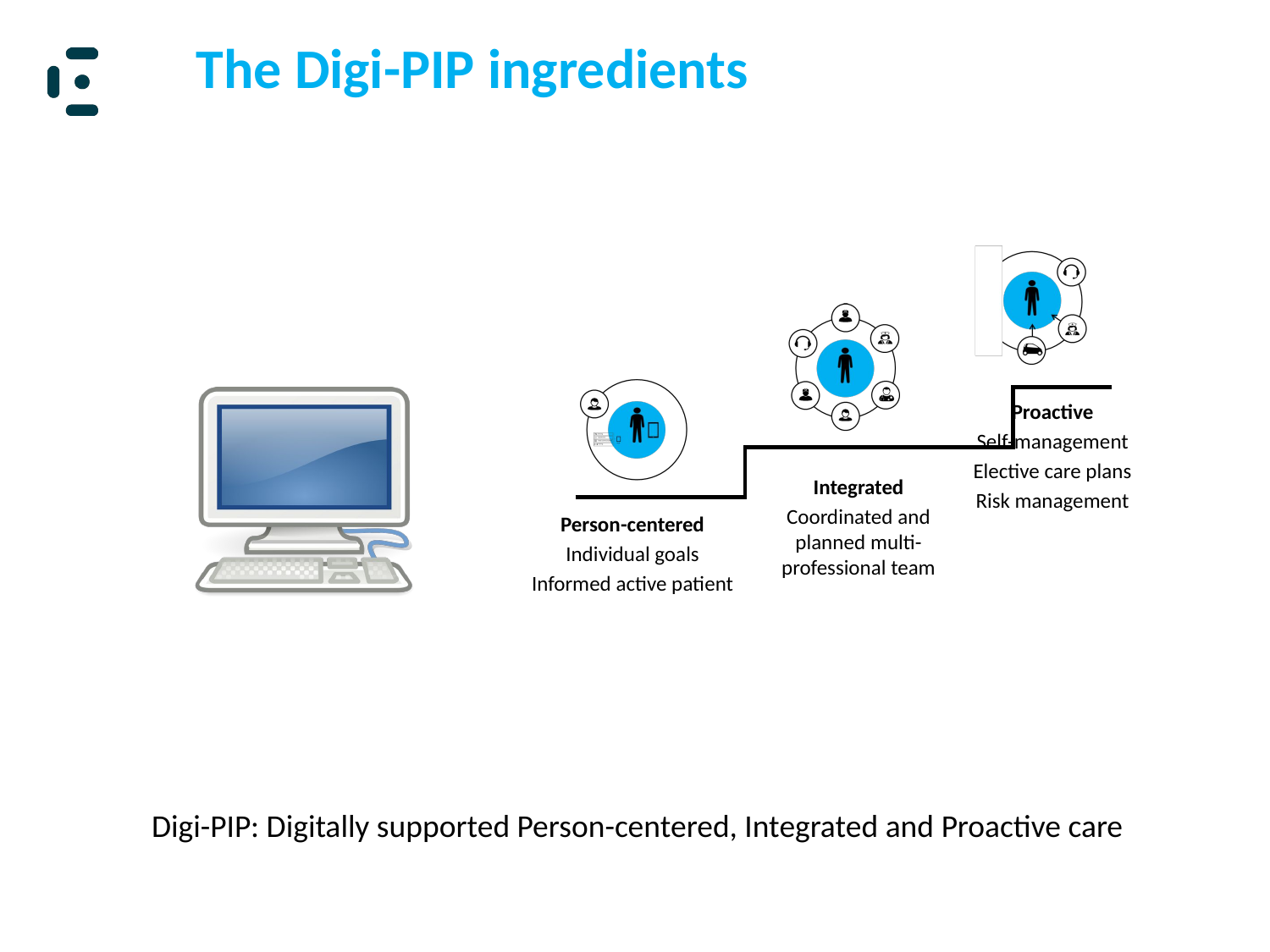

# The Digi-PIP ingredients
Proactive
Self-management
Elective care plans
Risk management
Integrated
Coordinated and planned multi-professional team
Person-centered
Individual goals
Informed active patient
Digi-PIP: Digitally supported Person-centered, Integrated and Proactive care

## Slide 4
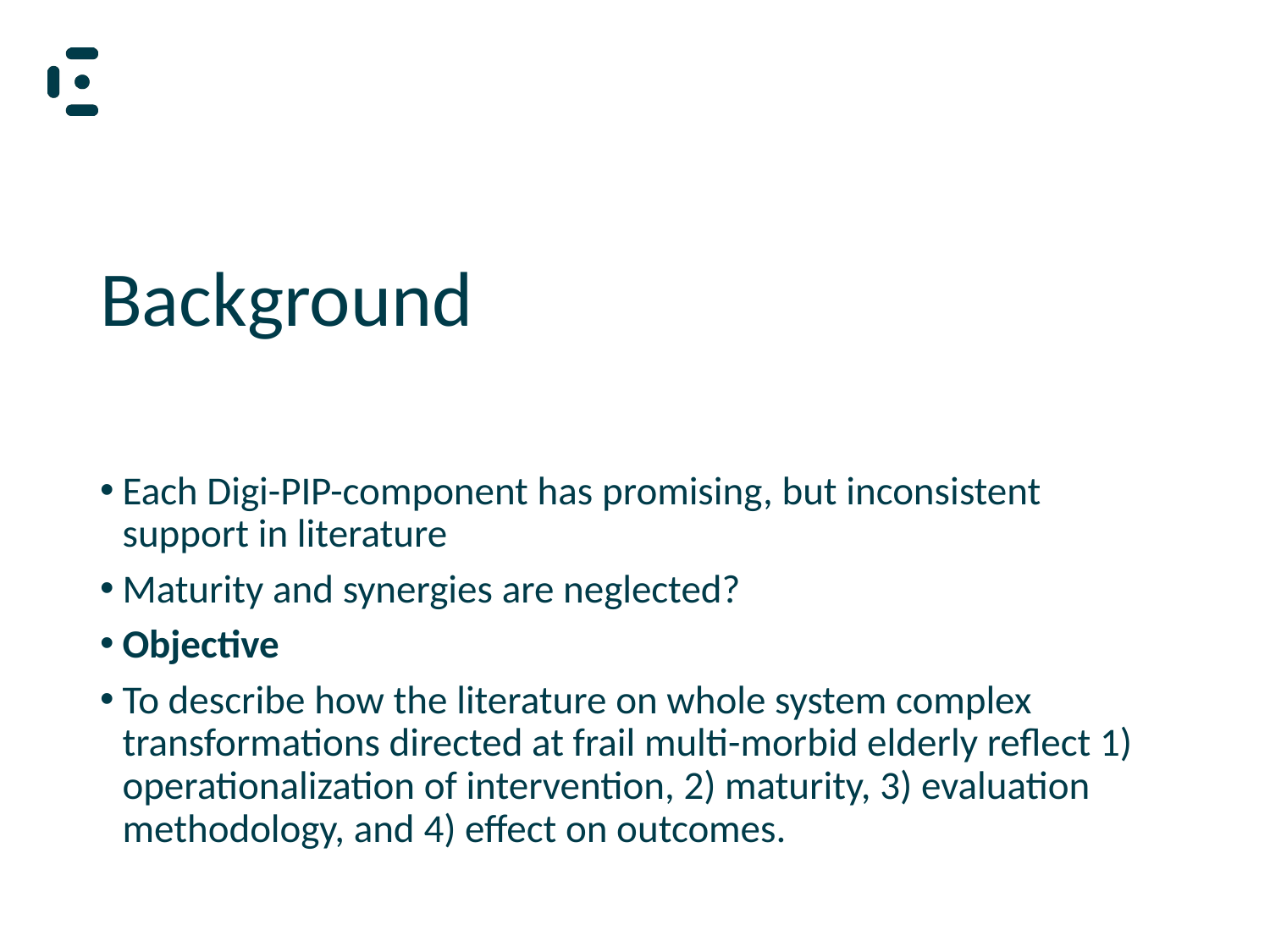

# Background
Each Digi-PIP-component has promising, but inconsistent support in literature
Maturity and synergies are neglected?
Objective
To describe how the literature on whole system complex transformations directed at frail multi-morbid elderly reflect 1) operationalization of intervention, 2) maturity, 3) evaluation methodology, and 4) effect on outcomes.

## Slide 5
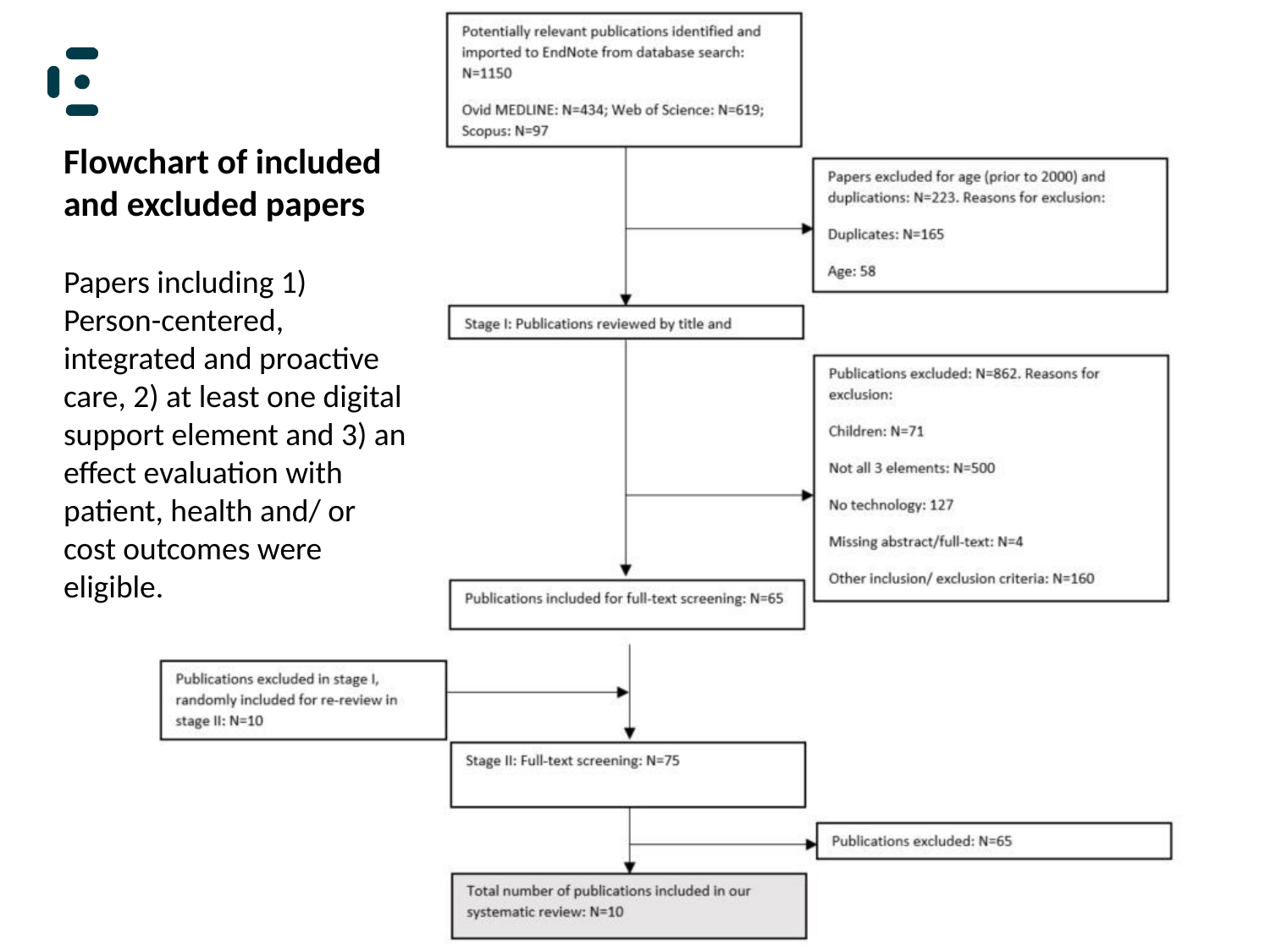

Flowchart of included and excluded papers
Papers including 1) Person-centered, integrated and proactive care, 2) at least one digital support element and 3) an effect evaluation with patient, health and/ or cost outcomes were eligible.

## Slide 6
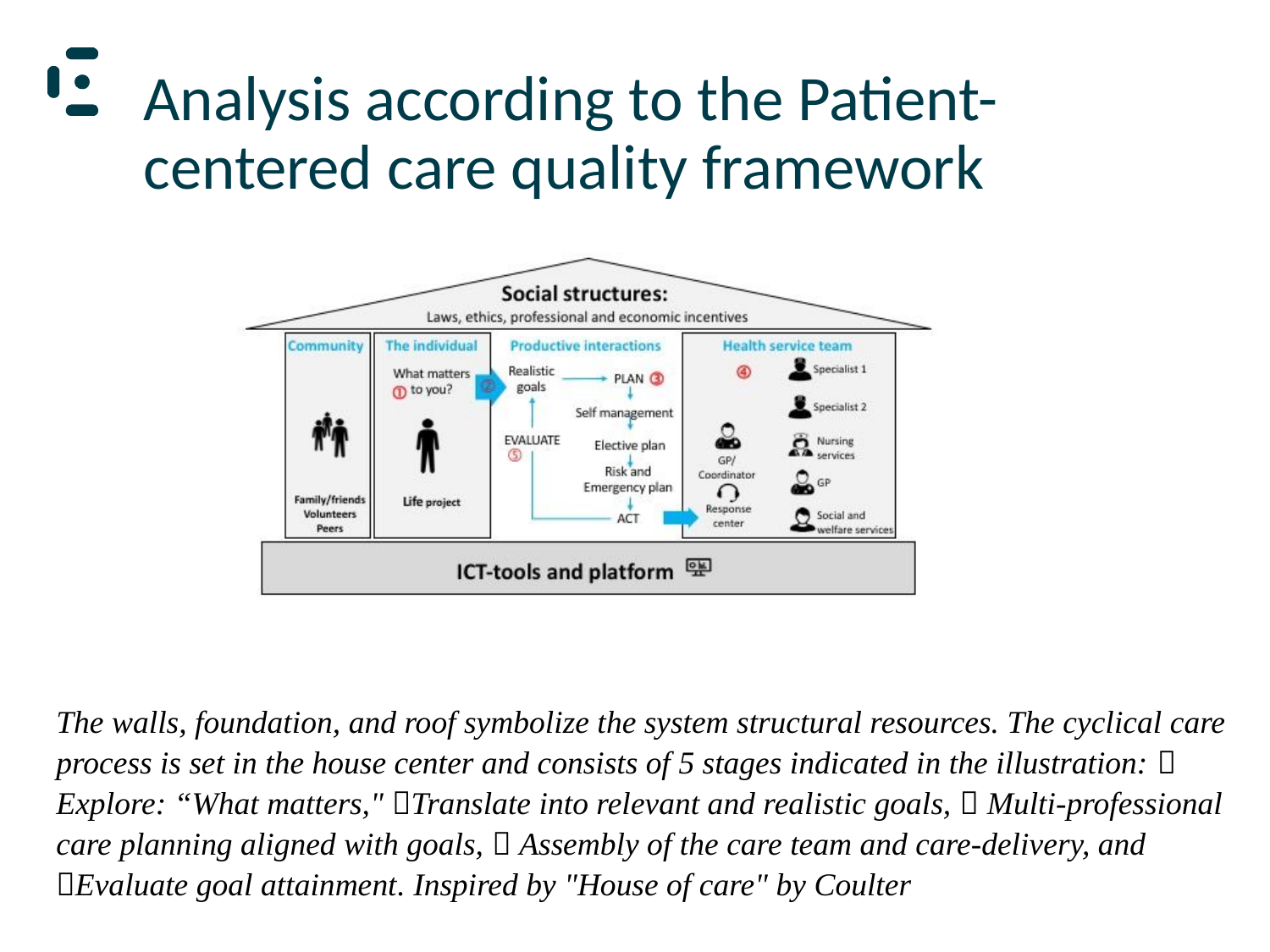

# Analysis according to the Patient-centered care quality framework
The walls, foundation, and roof symbolize the system structural resources. The cyclical care process is set in the house center and consists of 5 stages indicated in the illustration:  Explore: “What matters," Translate into relevant and realistic goals,  Multi-professional care planning aligned with goals,  Assembly of the care team and care-delivery, and Evaluate goal attainment. Inspired by "House of care" by Coulter

## Slide 7
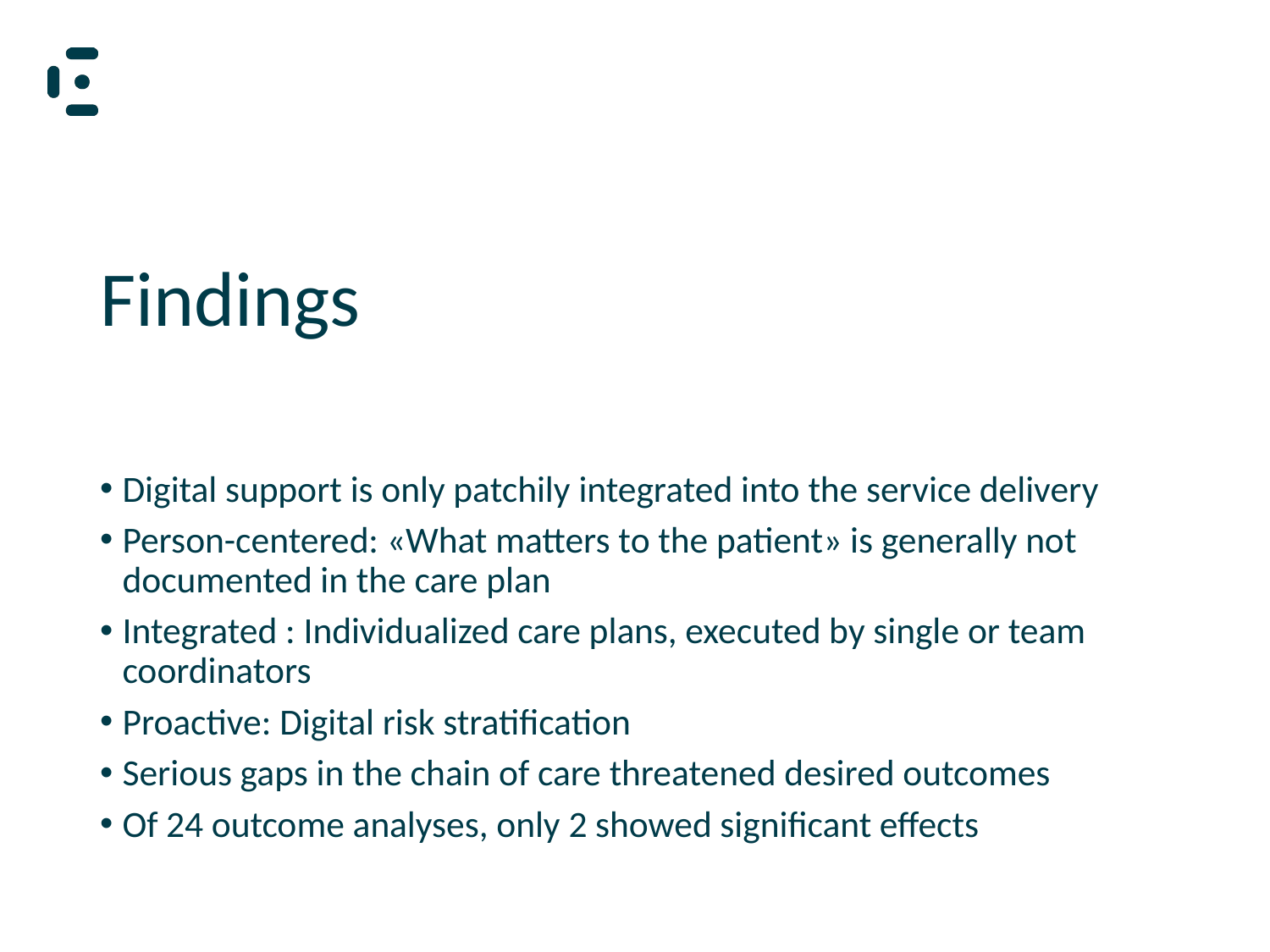

# Findings
Digital support is only patchily integrated into the service delivery
Person-centered: «What matters to the patient» is generally not documented in the care plan
Integrated : Individualized care plans, executed by single or team coordinators
Proactive: Digital risk stratification
Serious gaps in the chain of care threatened desired outcomes
Of 24 outcome analyses, only 2 showed significant effects

## Slide 8
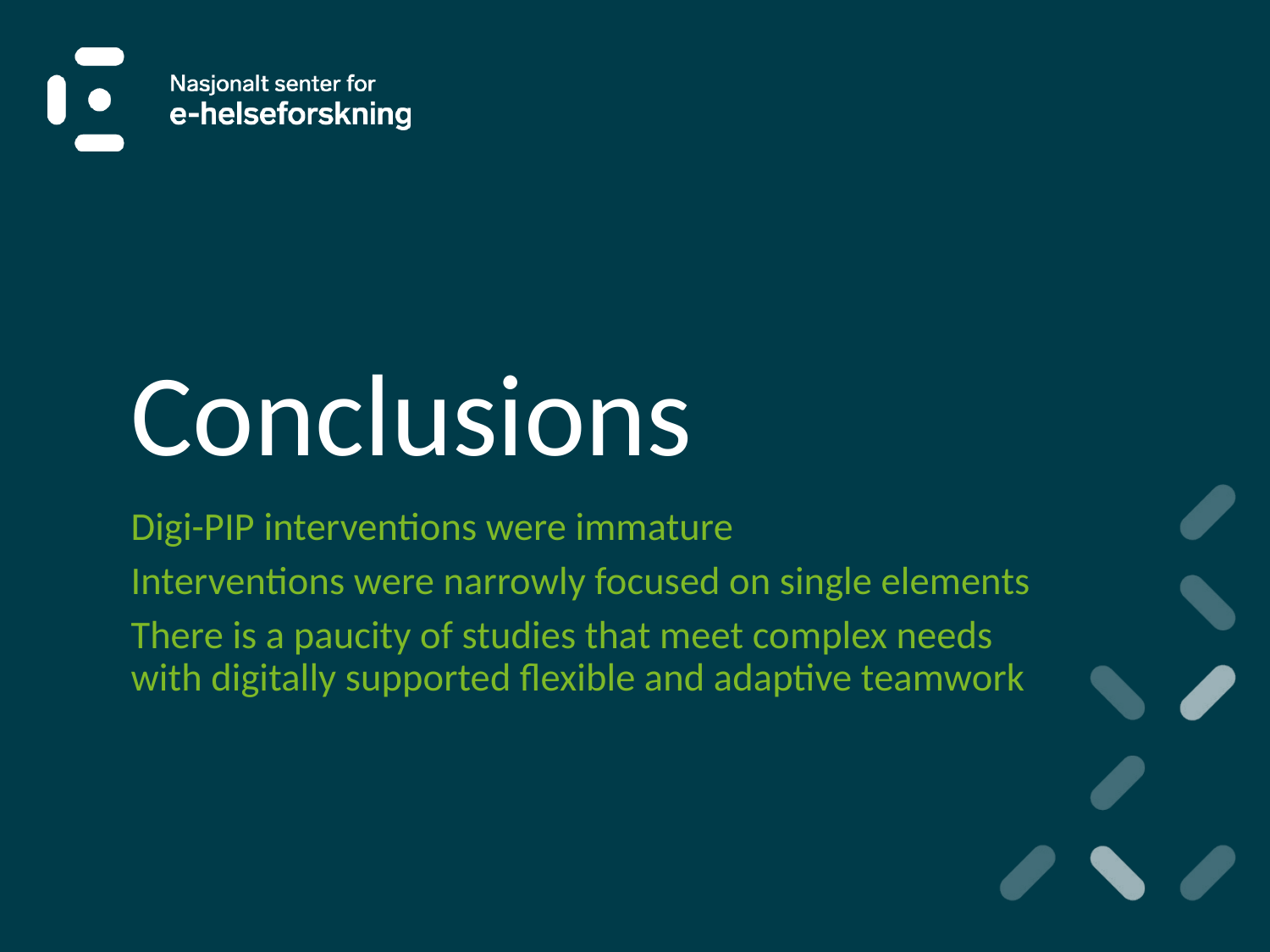

# Conclusions
Digi-PIP interventions were immature
Interventions were narrowly focused on single elements
There is a paucity of studies that meet complex needs with digitally supported flexible and adaptive teamwork
